# Meat Quality Characteristics of Mongolian Horses in Inner Mongolia: Regional Superiority and Transcriptomic Insights into Tenderness Differences Between Muscular Locations

**DOI:** 10.3390/ani16121788

**Published:** 2026-06-09

**Authors:** Yu Liu, Xuejiao Wang, Gesi Tan, Manglai Dugarjaviina, Xinzhuang Zhang

**Affiliations:** 1 Equus Research Center, College of Animal Science, Inner Mongolia Agricultural University, Hohhot 010000, China; liuyu97@nmgnydxwxy.wecom.work (Y.L.); 15024986715@163.com (G.T.); dmanglai@163.com (M.D.); 2 Rural Revitalization Research Institute, Inner Mongolia Agricultural University, Hohhot 010018, China; wangxuejiao881019@163.com

**Keywords:** Mongolian horses, meat quality, muscle location, fatty acids, transcriptome

## Abstract

Horse meat is a high-quality protein source. A total of 50 two-year-old male horses with similar body weights (300 ± 50 kg) were used: 30 for regional comparison (10 each from Bulgan province region of Mongolia (BPM), Tuv province region of Mongolia (TPM), and Inner Mongolia region of China (IMC)) and an additional 20 from the IMC region for muscle-site analysis. To elucidate the molecular mechanisms underlying regional and muscle-site differences in the meat quality of Mongolian horses, with particular emphasis on tenderness, this study analyzed chemical composition, minerals, fatty acids, and transcriptomes. Results showed that Mongolian horses from the Inner Mongolia region of China (IMC) had the highest contents of Se, C17:1, C18:2n6c, C18:3n3, ∑PUFA, PUFA/SFA, ∑n-3PUFA, ∑n-6PUFA (*p* < 0.05), and Fe (*p* < 0.01), and the lowest levels of C15:0 (*p* < 0.01) and C16:0 (*p* < 0.05). The Mongolian horses from the Tuv province region of Mongolia (TPM) had the highest Ca content (*p* < 0.05). The Mongolian horses from the Bulgan province region of Mongolia (BPM) had the highest ∑SFA (*p* < 0.01). The forelimb (FL) had the highest a*_45min_, b*_45min_ (*p* < 0.01), Fe, Zn, and C17:1 (*p* < 0.05), and the lowest shear force and drip loss (*p* < 0.01). The HD had the highest pH_45min_ and cooking loss (*p* < 0.01). The hindlimb (HD) increased the L*_45min_ compared with the FL (*p* < 0.01). Transcriptomic analysis identified 513 differentially expressed genes (DEGs) between FL and HD, including *SLC16A7*, *GPAM*, *FABP3*, and *TNNC1*. KEGG enrichment analysis revealed that these DEGs were significantly enriched in the cGMP-PKG signaling pathway, glycerophospholipid metabolism, and PPAR signaling pathway. In summary, this study demonstrated that among Mongolian horses from three different regions, IMC horses exhibited superior meat quality and flavor characteristics, and the FL of IMC horses showed significantly better meat quality than other anatomical sites. Transcriptomic analysis identified a set of candidate genes related to meat quality and lipid metabolism, including *SLC16A7*, *GPAM*, *FABP3*, and *TNNC1*, providing a scientific basis for further understanding of muscle-specific molecular mechanisms in Mongolian horses. Furthermore, the observed differences in physicochemical and nutritional properties across regions and muscle sites established a systematic foundation for quality grading, targeted nutritional utilization, and processing optimization of Mongolian horse meat.

## Simple Summary

Horse meat is becoming a popular healthy protein source. In this study, we examined meat from Mongolian horses, a hardy breed known for good meat quality. Our goal was to understand what affects meat quality, especially tenderness. First, we compared meat from three regions: two in Mongolia and one in Inner Mongolia, China. Inner Mongolian meat had higher levels of healthy fats and minerals (e.g., iron and selenium). Next, we focused on Inner Mongolian horses and compared forelimb and hindlimb meat. Forelimb meat was more tender, had better color, and lost less water during cooking. To explore the underlying reasons, transcriptome analysis revealed several genes involved in fat storage and muscle fiber type that were significantly upregulated in the tender forelimb meat, which likely contributed to the improved tenderness. Our findings can help select for better meat quality, benefiting both the industry and consumers who want tasty, nutritious food.

## 1. Introduction

Horse meat has gained increasing prominence in the global market as a high-quality protein source, characterized by its low fat content, high protein levels, and a rich profile of minerals and unsaturated fatty acids (FA) [1]. The Mongolian horse, a premier indigenous breed from the northern grasslands of China, is renowned for its exceptional resilience and adaptability to coarse fodder. Its meat is highly valued for its superior physicochemical properties and functional nutrients, making this breed a vital germplasm resource for the development of the specialized horse meat industry [2]. Meat quality is a complex trait modulated by various factors, including breed, age, geographical region, and anatomical muscle location [3]. Variations in ecological environments and husbandry practices across regions significantly influence the nutritional profile of meat, particularly its FA composition and mineral content [4]. For instance, Ivankovi’c et al. [5] reported that the meat of Croatian Posavina horses reared under the semi-extensive grazing system in the floodplain areas of the Sava River basin had a higher content of ω-3 polyunsaturated fatty acid (ω-3 PUFA), with α-linolenic acid (C18:3n3) reaching 6.06 g/100 g of total lipids. Additionally, the meat was rich in volatile aromatic compounds, exhibiting high tenderness and an excellent flavor profile. In comparison with intensively reared European heavy horse breeds (Italian Heavy Draught horse and Heavy French breeds), the meat of Croatian Posavina horses had a low intramuscular fat content of only 3.61% with a thin fat layer, and a nutritionally balanced ω-6/ω-3 ratio of 3.46. It was characterized by tender and juicy texture, and possessed both a unique sensory flavor and superior nutritional quality. Furthermore, due to distinct physiological functions and anatomical positions, muscles from different sites vary remarkably in muscle fiber type distribution, connective tissue content, and metabolic profiles. These variations ultimately dictate key palatability traits, such as meat color, tenderness, and water-holding capacity [3]. Among these, tenderness is the primary determinant of consumer acceptance. Its development is intricately linked to biological processes such as muscle fiber type transformation, intramuscular fat (IMF) deposition, and post-mortem myofibrillar protein degradation—all of which are governed by the differential expression of specific regulatory genes [6].

Transcriptome sequencing has emerged as a robust tool for elucidating the molecular mechanisms underlying meat quality traits and has been extensively applied to livestock such as cattle, sheep, and pigs [7,8,9]. For example, transcriptomic profiling of different muscles in Kazakh horses highlighted the pivotal role of pathways related to muscle fiber development and lipid metabolism in quality regulation [10]. Similarly, studies on Qinchuan cattle identified candidate genes associated with muscle fiber type switching, providing molecular targets for meat quality improvement [11]. However, systematic research on variations in muscle quality of Mongolian horses across different regions and anatomical locations remains scarce, and in particular, the molecular mechanisms governing muscle tenderness have not yet been fully elucidated.

Although scientific reports on horse meat quality are available, most studies have focused only on the effects of external factors such as sex, breed, and diet, and have commonly used the longissimus dorsi muscle as a representative sample to evaluate the impact of these factors on meat quality traits. However, systematic investigations into the quality differences among other muscles or cuts, which account for a substantial proportion of carcass weight and determine the final economic value of the carcass, remain limited. Furthermore, variations in rearing practices, geographical environment, and climatic conditions may lead to significant differences in the meat quality of Mongolian horses across regions, and understanding these regional differences is important for enhancing market competitiveness and guiding consumer choices. Therefore, to promote the consumption and trade of horse meat, more detailed research on the regional and anatomical factors affecting meat quality is urgently needed. In this context, the present study primarily investigated the effects of geographical origin and anatomical muscle site on the meat quality of Mongolian horses. We first evaluated the chemical composition, mineral content, and fatty acid profiles of muscles from Mongolian horses originating from different regions to systematically analyze the influence of geographical factors. Based on these findings, horses from the region exhibiting the optimal nutritional composition were selected for a detailed comparison of different anatomical muscle sites, aiming to define location-specific quality characteristics. Subsequently, focusing on muscles with the most pronounced differences in tenderness, transcriptome sequencing was employed to identify tenderness-related differentially expressed genes (DEGs) and to reveal the key genes and core signaling pathways driving these variations. This study aims to systematically characterize the regional- and muscle-specific molecular mechanisms underlying the meat quality of Mongolian horses, thereby providing a scientific foundation and data support for high-quality meat production, genetic improvement, and industrial development of this unique equine resource.

## 2. Materials and Methods

### 2.1. Animals

**Figure 1:**
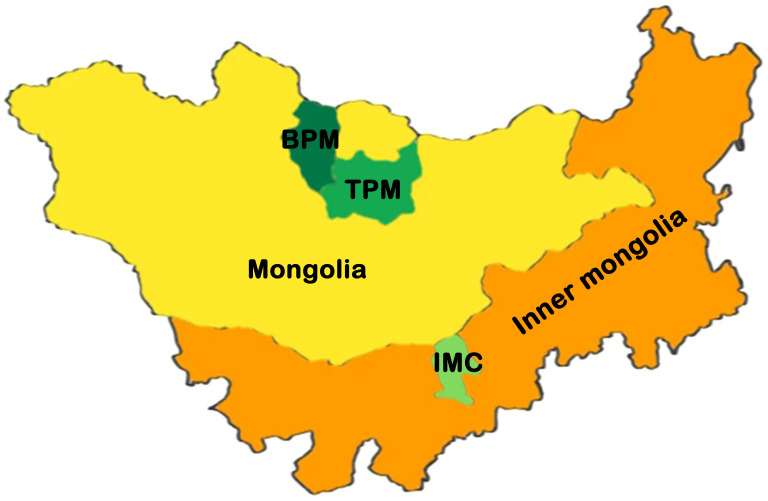
Geographic locations of the sampling regions for Mongolian horses. BPM: Bulgan Province, Mongolia; TPM: Tuv Province, Mongolia; IMC: Baotou, Inner Mongolia, China.

The Mongolian horses selected for this study originated from Bulgan province, Mongolia (BPM); Tuv province, Mongolia (TPM); Baotou in Inner Mongolia, China (IMC), respectively (Figure 1).

### 2.2. Experimental Design and Sample Collection

A total of 30 two-year-old male horses with similar body weights (300 ± 50 kg) were selected from three regions: BPM, TPM, and IMC, with ten from each region. Following a 24 h fast, the animals were electrically stunned and exsanguinated at a commercial slaughterhouse in compliance with European Commission welfare regulations. Immediately post-slaughter, samples were collected from the longissimus thoracis (LT) and were kept in liquid nitrogen for chemical, mineral, and FA composition analysis. After fully freeze-drying the samples of LT using a lyophilizer (Lyolab, Coolvacuum, Spain), the contents of chemical, mineral, and FA were determined.

For the muscle-site analysis, twenty additional two-year-old male horses (independent from the 30 horses used for the regional analysis) were sourced from the same IMC region (Baotou, China). These 20 horses had identical origins, similar rearing conditions, and comparable body weight to the first cohort. Following a 24 h fast, the animals were electrically stunned and exsanguinated at a commercial slaughterhouse in compliance with European Commission welfare regulations. Immediately post-slaughter, samples were collected from the forelimb (FL), LT, and hindlimb (HD). After removing fascia and connective tissue, part of the samples were analyzed for pH and meat color. Subsequently, samples were either vacuum-sealed and stored at 4 °C for quality assessment, and part of the samples snap-frozen in liquid nitrogen for nutritional and transcriptomic analyses. A portion of the samples was lyophilized (Lyolab, Coolvacuum, Spain) to determine protein, fat, ash, FA, and mineral content, while the remainder was stored at −80 °C. All procedures were approved by the Animal Care and Use Committee of Inner Mongolia Agricultural University (No. NND2024009).

### 2.3. Meat Quality Analysis

Meat quality parameters were evaluated according to Ivanković et al. [5]. The initial pH value (pH_45min_) and meat color (L*_45min_, a*_45min_, b*_45min_) were recorded 45 min after slaughter using a Testo 205 portable pH meter (Testo Instrument International Trading (Shanghai) Co., Ltd., Shanghai, China) and a CR-400 colorimeter (Konica Minolta (China) Investment Co., Ltd., Shanghai, China), respectively, with each muscle sample analyzed in triplicate. Cooking loss was determined according to the following method: approximately 120 g of muscle tissue samples were weighed (W1), vacuum-sealed in retort pouches, and heated in a constant temperature water bath at 80 °C. A food core thermometer was used to confirm that the core temperature of the samples reached 70 °C. The pouches were then opened, surface moisture was blotted, and the samples were weighed again after cooling (W2). The cooking loss was calculated as [(W1 − W2)/W1] × 100%. All measurements were conducted in triplicate. For water loss rate, 2.523 cm diameter cores were pressed at 35 kg for 5 min between 18 layers of filter paper, with the weight difference expressed as a percentage. Shear force was measured on cooked samples using a C-LM3 tenderness tester (Nanjing Mingao Instrument Equipment Co., Ltd., Nanjing, China) by extracting 1.27 cm diameter cores parallel to the muscle fibers. All tests were performed in triplicate.

### 2.4. Determination of Chemical and Mineral Composition

Chemical constituents were quantified following Chinese National Standards (GB). Crude fat was determined via Soxhlet extraction with anhydrous diethyl ether (GB/T 5009.6-2016) [12]. Ash content was measured by incinerating 5 g samples in a muffle furnace (MFLC-16/12C, Tianjin Taisite Instrument Co., Ltd., Tianjin, China) at 550 °C for 8 h until a constant weight was achieved (GB/T 5009.4-2016) [13]. Crude protein was analyzed using the Kjeldahl method with Tecator Digestion System and Kjeltec Auto 1030 Analyzer (KT260, FOSS, Hillerød, Denmark) in accordance with GB 5009.5-2016 [14]. For mineral analysis, 5 g LT samples were pretreated following Seong et al. [3]. The samples were destroyed by dry ashing in a muffle furnace (MFLC-16/12C, Tianjin Taisite) for 12 h with a final temperature of 600 °C. The ash content was dissolved in 10 mL of 37% HCl and distilled water (1:1 *v*/*v*) solution, filtered through filter paper, and then diluted to a final volume of 10 mL with distilled water. Calcium (Ca, 422.7 nm), iron (Fe, 248.3 nm), zinc (Zn, 213.9 nm), and selenium (Se, 196.0 nm) were detected via ICP-OES (ZEEnit700P, Analytik Jena, Jena, Germany). Distilled water was used as the blank solution. Each sample was measured three times, and the mean values were used for analysis.

### 2.5. Fatty Acid Profile

Fatty acid profiles were determined and analyzed with reference to the national standard GB 5009.168-2016 [15] and the method described by Wu et al. [16] with minor modifications. Briefly, each meat sample (25 mg) was slowly thawed at 4 °C, mixed with 5 mL of dichloromethane-methanol (2:1, *v*/*v*), vortexed, and then 2 mL of gold-labeled water was added. After washing and phase separation, the organic layer was collected and evaporated to dryness under nitrogen. The residue was reconstituted in 2 mL of n-hexane, spiked with an internal standard, and subjected to methylation for 0.5 h. Following a second wash with 2 mL of ultrapure water, 2000 μL of the supernatant were transferred, dried under nitrogen, and re-dissolved in n-hexane. The final extract was transferred to an injection vial for GC–MS analysis.

GC–MS analysis was performed on a system consisting of an Agilent 7890B gas chromatograph coupled with an Agilent 5977B MSD mass spectrometer (Agilent Technologies, Inc., Santa Clara, CA, USA). Separation was achieved on an Agilent HP-5ms capillary column (30 m × 250 μm × 0.25 μm). The oven temperature program was as follows: initial 80 °C, then ramped to 180 °C at 20 °C/min and held for 8 min, and finally increased to 280 °C at 5 °C/min and held for 3 min. Helium was used as the carrier gas at a flow rate of 1.0 mL/min. The injection volume was 1 μL with a split ratio of 10:1. Mass spectrometric detection was performed in electron impact (EI) mode at 70 eV, using a combination of full scan and selected ion monitoring (SIM) modes. The ion source temperature was 230 °C, the transfer line temperature was 250 °C, and the inlet temperature was 280 °C. MSD ChemStation software (Version E.02.00.493) was used to extract the chromatographic peak area and retention time. Draw a calibration curve and calculate the content of fatty acids in the sample.

### 2.6. Transcriptomics Analysis

FL and HD samples were sent to Inner Mongolia Benniu Technology Co., Ltd. (Hohhot, China) for transcriptome sequencing. Total RNA was isolated and purified from FL and HD samples using TRIzol reagent (Invitrogen, Carlsbad, CA, USA). The quantity and purity of total RNA were determined by NanoDrop ND-1000 (NanoDrop, Wilmington, DE, USA). RNA integrity was further assessed via Bioanalyzer 2100 (Agilent, Santa Clara, CA, USA), and libraries were constructed following agarose electrophoresis protocols. Finally, paired-end sequencing was performed on an Illumina Novaseq™ 6000 platform (LC Bio Technology Co., Ltd., Hangzhou, China) with a PE150 sequencing mode.

Raw reads were processed using Cutadapt (version 1.9) to remove adapter sequences, low-quality bases, and undetermined nucleotides. The cleaned reads from each sample were then aligned to the equine reference genome (Equus caballus, version EquCab3.0), which is publicly accessible via the Ensembl database (http://www.ensembl.org/Equus_caballus/Info/Index, accessed on 10 January 2025). Aligned reads were assembled into transcripts using StringTie, and transcriptomes from all samples were merged using gffcompare. Gene expression levels were quantified as fragments per kilobase of transcript per million mapped reads (FPKM).

Differential expression analysis between samples was conducted using the R packages edgeR (https://bioconductor.org/packages/release/bioc/html/edgeR.html, accessed on 9 June 2026) or DESeq2 (http://www.bioconductor.org/packages/release/bioc/html/DESeq2.html, accessed on 9 June 2026). Differentially expressed genes (DEGs) were defined as those with a fold change (FC) ≥ 2 or FC ≤ 0.5 (i.e., |log2FC| ≥ 1) and a *p*-value < 0.05. These criteria were applied to identify up-regulated and down-regulated genes between sample groups. The resulting DEGs were subsequently subjected to GO and KEGG enrichment analyses. The raw sequencing data supporting the conclusions of this article will be made available by the authors on request, but the raw sequence data is unavailable due to file damage.

### 2.7. Total RNA Extraction, cDNA Synthesis, and Fluorescence Quantitative PCR Detection

To verify the reliability of the transcriptomic data, six genes were randomly selected for expression analysis following the methodology of Zhang et al. [17]. Total RNA was extracted from the FL and HD samples using Trizol reagent (Tiangen Biochemical Technology Co., Ltd., Beijing, China) according to the manufacturer’s protocol. Briefly, the FL and HD samples were thoroughly homogenized in 1 mL of Trizol using a sterile pestle, after which 0.2 mL of chloroform was added. The mixture was vigorously vortexed for 15 s, incubated at room temperature for 3 min, and then centrifuged at 12,000× *g* for 15 min at 4 °C. The upper aqueous phase was carefully transferred to a new RNase-free centrifuge tube, and an equal volume of isopropanol was added to precipitate the RNA by incubating at room temperature for 10 min. The RNA pellet was collected by centrifugation at 12,000× *g* for 10 min at 4 °C, rinsed with 1 mL of 75% ethanol, air-dried at room temperature, and finally dissolved in 30 μL of double-distilled water (ddH2O). Subsequently, first-strand cDNA was synthesized using the Evo M-MLV Reverse Transcription Premix Kit (Accurate Biology, Hangzhou, China). The mRNA expressions of glyceraldehyde-3-phosphate dehydrogenase (*GAPDH*), homeobox C10 *(HOXC10*), homeobox D8 (*HOXD8*), LIM domain kinase 2 (*LIMK2*), corticotropin releasing hormone receptor 2 (*CRHR2*), glycerol-3-phosphate acyltransferase, mitochondrial (*GPAM*) and protein phosphatase, Mg^2+^/Mn^2+^ dependent 1J (*PPM1J*) were determined by RT-qPCR using a BioRad CFX96 Real-Time PCR system (Bio-Rad Laboratories, Hercules, CA, USA) (Table 1). The amplification program consisted of an initial denaturation at 95 °C for 10 s, followed by 40 cycles of 95 °C (10 s), 60 °C (30 s), and 72 °C (60 s). GAPDH was used as the reference gene. Relative quantification was performed using the 2^−ΔΔCT^ method.

### 2.8. Statistics

Experimental data were initially processed and formatted using Microsoft Excel. After normality was confirmed using the Shapiro–Wilk test, meat quality, chemical and mineral composition, and FA were assessed by one-way ANOVA using SPSS 26.0. When effects were significant (*p* < 0.05), Tukey’s multiple range test was used to identify significant differences among the categories. GraphPad Prism 8 (GraphPad Software, La Jolla, CA, USA) was used to visualize the data by expressing the mean and standard error of the mean (SEM). All data are presented in tables as mean  ±  SEM.

## 3. Results

### 3.1. Differences in Chemical and Mineral Composition Among Mongolian Horses from Different Regions

Table 2 showed that the IMC had the highest Fe (*p* < 0.01) and Se (*p* < 0.05) compared with the BPM and TPM. Compared with the BPM and IMC, the TPM induced a higher Ca (*p* < 0.05). There were no significant differences in the contents of protein, fat, and ash in the DM of muscles from different regions of Mongolian horses (*p* > 0.05).

### 3.2. Differences in FA Composition Among Mongolian Horses from Different Regions

Table 3 showed that the IMC had the highest C17:1, C18:2n6c, C18:3n3, ∑PUFA, PUFA/SFA, ∑n-3PUFA, ∑n-6PUFA (*p* < 0.05), and had the lowest C15:0 (*p* < 0.01) and C16:0 (*p* < 0.05) compared with the BPM and TPM. The BPM had the highest ∑SFA (*p* < 0.01) compared with the IMC and TPM. Among saturated fatty acids (SFA), the most abundant ones were C16:0 and C18:0, accounting for more than 80% of the total SFA content. Among monounsaturated fatty acids (MUFA), the most abundant ones were C18:1n9c. The n-6/n-3 PUFA ratio was 1.79–1.87, which was within the reasonable range of 1.5–2.0.

### 3.3. Differences in Meat Quality Among Different Muscle Parts of Mongolian Horses

Table 4 showed that the FL had the highest a*_45min_ and b*_45min_, and the lowest shear force and water loss compared with the HD and LT (*p* < 0.01). Compared with the FL and LT, HD had the highest pH_45min_ and cooking loss (*p* < 0.01). The HD increased the L*_45min_ compared with the FL (*p* < 0.01).

### 3.4. Differences in Chemical and Mineral Composition Among Different Muscle Parts of Mongolian Horses

Table 5 showed that the FL had the highest Fe and Zn compared with the LT and HD (*p* < 0.05). There were no significant differences in the contents of protein, fat, and ash in the DM of muscles from different parts of Mongolian horses (*p* > 0.05).

### 3.5. Differences in FA Composition Among Different Muscle Parts of Mongolian Horses

Table 6 showed that FL had the highest C17:1 compared with the HD and LT (*p* < 0.05). Among SFA, the most abundant ones were C16:0 and C18:0, accounting for more than 80% of the total SFA content. Among MUFA, the most abundant ones were C18:1n9c. The n-6/n-3 PUFA ratio was 1.73–1.99, which was within the reasonable range of 1.5–2.0.

### 3.6. Transcriptomics Analysis

**Figure 2:**
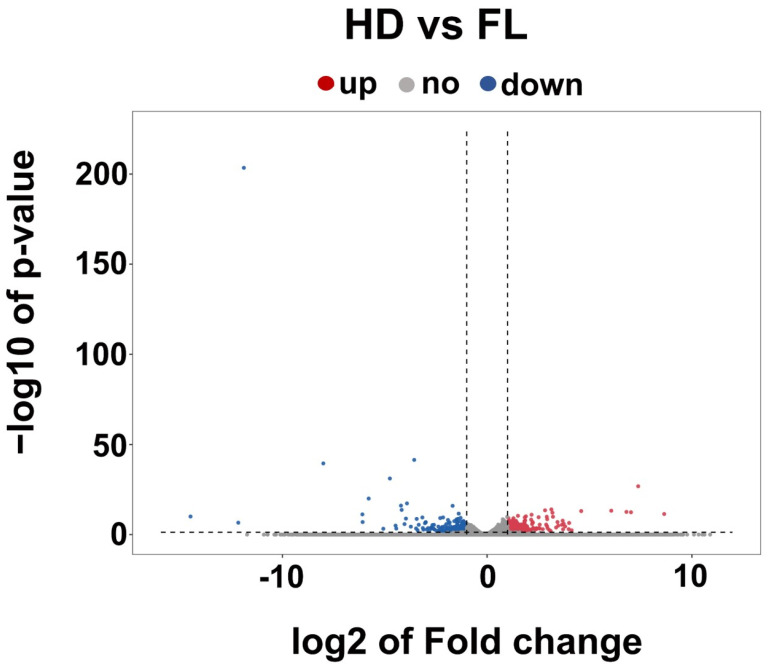
Volcano plot of DEGs in the HD vs. FL comparison. n = 8.

To compare the difference in muscle mRNA between the HD and FL groups, the thresholds of *p*-value < 0.05 and FC > 2 or FC < 0.5 were used to identify the differentially expressed genes. In this study, a total of 513 DEGs were identified between the HD and FL groups (*p* < 0.05). Compared with the HD group, 266 genes were up-regulated (*SLC16A7*, *GPAM*, *FABP3*, etc.) and 247 genes were down-regulated (*DDI2*, *PLEC*, *DST*, etc.) in the FL group (Figure 2; Table 7).

**Figure 3:**
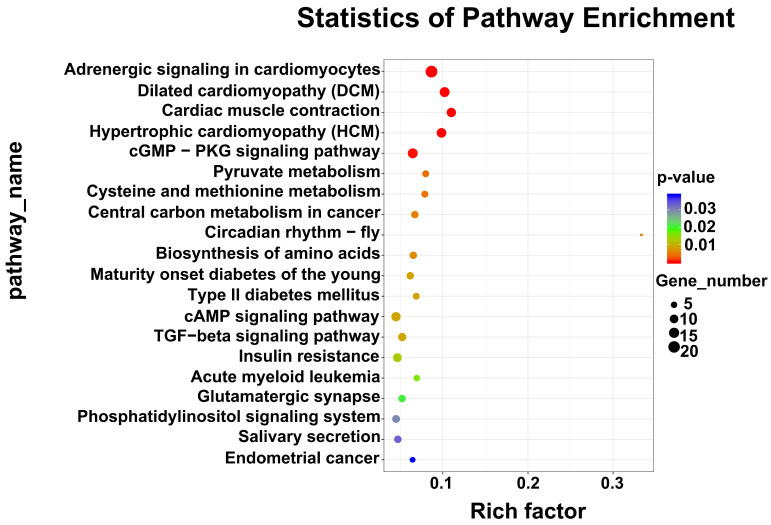
KEGG pathway enrichment analysis of up-regulated and down-regulated DEGs. n = 8.

KEGG pathway enrichment analysis revealed that the DEGs were significantly enriched in 25 pathways (*p* < 0.05), including “Adrenergic signaling in cardiomyocytes”, “Cardiac muscle contraction”, “Glycerophospholipid metabolism”, “PPAR signaling pathway”, “Calcium signaling pathway”, and “cGMP-PKG signaling pathway”. A bubble plot was generated to display the top 20 most significantly enriched pathways (Figure 3). Among these, pathways potentially associated with meat quality traits included “cGMP-PKG signaling pathway”, “Glycerophospholipid metabolism”, and “PPAR signaling pathway”.

### 3.7. qRT-PCR Analysis

**Figure 4:**
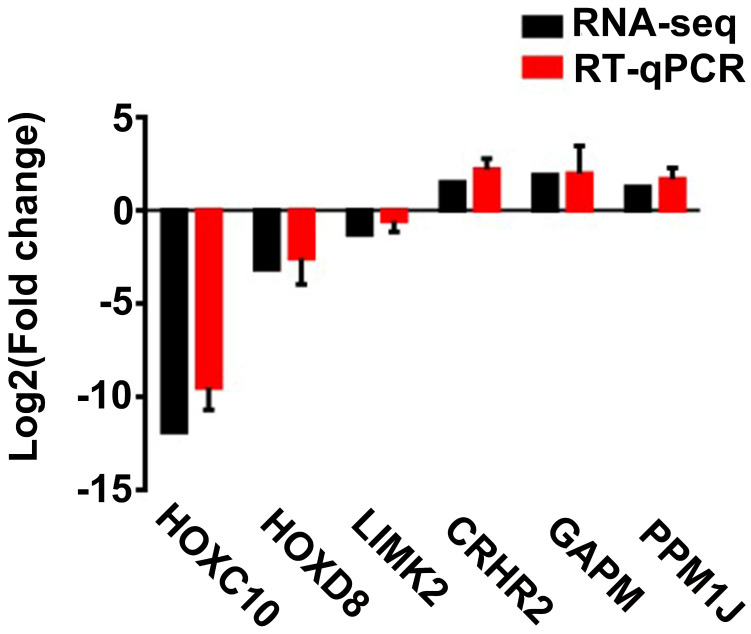
Validation of DEGs. n = 8.

To verify the transcriptome sequencing data, six genes (*HOXC10*, *HOXD8*, *LIMK2*, *CRHR2*, *GPAM*, and *PPM1J*) were randomly selected for validation using qRT-PCR. As shown in Figure 4, the expression trends of the detected genes were consistent between the RNA-Seq and qRT-PCR results, indicating that the sequencing data are reliable.

## 4. Discussion

Fatty acids in muscle are among the major factors influencing meat quality [18]; their content and composition affect meat color, juiciness, flavor, and nutritional value, among other attributes [19]. Saturated fatty acids (SFAs) are negatively correlated with flavor, and myristic acid (C14:0) and palmitic acid (C16:0) are considered detrimental to human health [20]. In this study, the levels of C15:0 and C16:0 in horse muscle from the BPM and TPM regions were significantly higher than those from the IMC region. Differences in muscle flavor mainly arise from fat decomposition and oxidation [21]. During oxidation, polyunsaturated fatty acids present in lipid-dissolved muscle fiber bundles are thought to improve meat quality and generate flavor compounds such as aldehydes, ketones, alcohols, esters, and aliphatic compounds through a series of oxidative reactions [22]. These compounds participate in the Maillard reaction, in which olefinic aldehyde compounds are especially important precursors for the production of aromatic substances in muscle [23]. In this study, the contents of C17:1, C18:2n-6c, C18:3n-3, ∑PUFA, ∑n-3PUFA, and ∑n-6PUFA, as well as the PUFA/SFA ratio, in horse muscle from the IMC region were significantly higher than those from the BPM and TPM regions. This difference suggests that horse meat from the IMC region may possess a more desirable flavor profile. Overall, in terms of fatty acid composition and content, horses from the IMC region exhibited more favorable characteristics compared with those from the BPM and TPM regions. Minerals are essential nutrients that play a critical role in both human and animal health, influencing a range of physiological functions including enzymatic activity, osmotic regulation, and muscle contraction [24]. They also contribute significantly to meat quality by affecting multiple biological processes in the animal, as well as postmortem attributes such as color and texture. For instance, Fe participates in the calpain system and lipid metabolism, thereby modulating tenderness and fatty acid composition [25]. Elevated Fe levels can promote intramuscular fat deposition and influence tenderness through upregulation of lipogenic genes (e.g., *SCD1*, *FASN*) and the mTOR signaling pathway [26]. Se is involved in selenoprotein synthesis and the expression of lipid-metabolism-related genes (e.g., *APOE*, *LPL*), and it regulates lipid metabolism and total lipid deposition in muscle through the modulation of thyroid hormone activity [27]. Ca acts as a key cofactor for calpains, affecting calpain activity and postmortem proteolysis, and thereby contributing to postmortem tenderization [28]. In the present study, horses from the IMC region exhibited significantly higher Fe and Se concentrations, but notably lower Ca levels, in muscle tissue compared with horses from the BPM and TPM regions.

Visual assessment of meat color remains a common method for evaluating freshness, whereas tenderness and juiciness substantially influence consumer purchasing behavior [29]. Muscle tenderness is a key indicator of meat quality, typically measured by shear force: lower shear force reflects finer muscle fibers and more tender meat with better texture [30]. Drip loss and cooking loss are also indicators of muscle water-holding capacity, affecting meat color, flavor, tenderness, and nutritional value. Meat with high water-holding capacity generally appears juicy, tender, and moist. In contrast, meat with low water-holding capacity may become dry and tough due to surface moisture exudation, loss of soluble nutrients, and flavor alteration, thereby reducing overall meat quality. Moreover, high cooking loss can make meat tough and chewy, leading to diminished flavor [31]. The present study revealed that the FL exhibited significantly lower shear force, drip loss, and cooking loss than the HL. Furthermore, significant differences were observed between the forelimb and hindlimb of Mongolian horses in pH and color parameters (L*, a*, b*). These findings are consistent with previous reports. Litwinczuk et al. [32] found that the pH of the longissimus dorsi muscle in 10-year-old horses (5.72) was significantly higher than that of the semitendinosus muscle (5.69). Seong et al. [33] further confirmed significant differences in L, a*, and shear force among different muscle regions in horses. As a postural muscle group with high contraction frequency, the forelimb possesses a greater density of slow-twitch (Type I) oxidative fibers and higher myoglobin concentration, which partly explains its superior meat color and tenderness. Additionally, its inherently smaller fiber diameter and more compliant, less dense connective tissue arrangement may also contribute to improved tenderness [3]. By contrast, the hindlimb is dominated by fast-twitch (Type II) glycolytic fibers, which are associated with extensive myofibrillar cross-linking and increased mechanical resistance, thereby potentially elevating shear force [34,35]. Li et al. [23] reported significant differences in muscle fiber characteristics among different muscle regions in Kazakh horses. Seong et al. [33] made similar observations regarding quality variations across anatomical locations in horse meat, noting that frequently active muscles exhibit superior tenderness and meat color attributable to a higher proportion of slow-twitch fibers—a mechanism indirectly supported by the present study. Flavor development is primarily determined by the amino acid profile, as amino acids serve as essential precursors to flavor compounds. The crude protein content of meat is fundamental to meat quality assessment, and its level is closely related to the content and composition of amino acids. Fatty acid composition and concentration modulate oxidative stability and sensory attributes while also playing a decisive role in nutritional value [36]. Excessive intake of SFAs is associated with elevated triglycerides and low-density lipoprotein levels, thereby increasing the risk of obesity, diabetes, and coronary heart disease. In contrast, monounsaturated and polyunsaturated fatty acids (PUFAs) help improve cardiovascular function and immune performance by lowering serum cholesterol [37]. In this study, no significant differences were detected in protein, fat, or ash contents among different muscle regions of Mongolian horses. However, the hindlimb showed higher levels of heptadecenoic acid (C17:1). This difference may be attributed to variations in triglyceride and phospholipid contents among muscle regions. Our findings are generally consistent with those of Li et al. [38], who demonstrated that muscle type significantly influences fatty acid composition. Among minerals commonly found in foods, Fe, manganese (Mn), and copper (Cu) are classified as trace elements essential for maintaining human health; inadequate intake of these trace minerals can lead to nutritional deficiency symptoms [39]. The present study found that the forelimb had the highest Fe and Zn contents. Similarly, Franco and Lorenzo [40] reported comparable levels of Cu, Fe, and Zn in various muscles of 15-month-old foals.

With the rapid progress of next-generation sequencing technologies, RNA-seq has emerged as a widely adopted approach in animal husbandry for investigating gene function and identifying novel genes [41]. A total of 513 DEGs were identified between the FL and HD. Among these, several DEGs (e.g., *SLC16A7*, *TNNC1*, *FABP3*, and *GPAM*) have been reported to be closely associated with meat quality traits such as intramuscular fat deposition, improved fatty acid composition, and increased tenderness. *SLC16A7* has been identified as a gene involved in triglyceride deposition [42]. Wang et al. [10] reported that *SLC16A7* expression in chicken skeletal muscle was positively correlated with triglyceride content and several FAs (C14:0, C16:0, C14:1, C16:1, and C18:1n9c) based on a genome-wide association study and weighted gene coexpression network analysis. Functional validation further demonstrated that *SLC16A7* promotes triglyceride accumulation in myocytes primarily through de novo lipogenesis, with a predominant role in muscle cells rather than adipocytes [10]. *GPAM* encodes a rate-limiting enzyme in the biosynthesis of triglycerides and phospholipids, catalyzing the initial step of triglyceride synthesis. Lee et al. [43] showed that miR-375 up-regulates *GPAM* expression by binding to its 3′-untranslated region, thereby promoting the expression of key adipogenic transcription factors (C/EBPα and PPARγ) and significantly increasing lipid droplet accumulation and triglyceride content in 3T3-L1 adipocytes. In addition, polymorphisms in the *GPAM* gene have been reported to be significantly associated with marbling scores in Hanwoo cattle, underscoring its potential role in IMF deposition [44]. *TNNC1* serves as the calcium-binding subunit of the troponin complex and plays a central role in regulating contraction in slow oxidative muscle fibers. In a proteomic study on foal meat quality, López-Pedrouso et al. [45] observed that TNNC1 abundance was negatively correlated with shear force, suggesting a close association with improved tenderness. Furthermore, an integrative multiomics analysis by Liu et al. [46] revealed that up-regulation of *TNNC* in Luchuan pigs significantly increased the proportion of slow oxidative muscle fibers, which was associated with superior meat quality traits, including a* value, water-holding capacity, tenderness, and marbling. *FABP3* functions as a key intracellular carrier for long-chain fatty acids. In muscle cells, it is responsible for transporting FAs from the plasma membrane to mitochondria for β-oxidation or directing them toward triglyceride and phospholipid synthesis [47]. In an integrated transcriptomic and metabolomic analysis of the *longissimus dorsi* muscle from Taihang Yun cattle, Chinese Simmental cattle, and Charolais cattle, Zhang et al. [48] observed that *FABP3* expression was significantly up-regulated in Taihang Yun cattle, a breed characterized by high IMF content. Among the three breeds, FABP3 protein abundance was highest in Taihang Yun cattle. Further gene-pathway-metabolite network analysis revealed that *FABP3* is closely associated with the PPAR and AMPK signaling pathways, which are recognized as central hubs regulating adipogenesis. Moreover, Malgwi et al. [19] reported that *FABP3* expression in porcine muscle is positively correlated with IMF content. By facilitating fatty acid uptake and intracellular transport, *FABP3* may promote IMF accumulation, thereby potentially improving meat tenderness, juiciness, and flavor. The enrichment of DEGs in pathways such as cGMP-PKG signaling, glycerophospholipid metabolism, and PPAR signaling suggests that these pathways could be involved in regulating intramuscular fat (IMF) deposition and tenderness [49,50,51]. The results of this study showed that the expression levels of *SLC16A7*, *GPAM*, *TNNC1*, and *FABP3* differed significantly between the FL and HD of Mongolian horses, with all four genes being markedly up-regulated in the FL relative to the HD. Based on these observations, we hypothesized that these genes may be involved in regulating meat quality traits. Importantly, no other data on genes controlling meat quality in Mongolian horses were currently available for direct comparison, and functional validation in equine muscle tissue was lacking in the present study. Therefore, this study primarily revealed correlations between gene expression and meat quality traits, whereas the specific causal regulatory mechanisms remained to be elucidated through future functional experiments.

## 5. Conclusions

In conclusion, the IMC had the highest contents of Se, C17:1, C18:2n6c, C18:3n3, ∑PUFA, PUFA/SFA, ∑n-3PUFA, ∑n-6PUFA (*p* < 0.05) and Fe (*p* < 0.01), while exhibiting the lowest levels of C15:0 (*p* < 0.01) and C16:0 (*p* < 0.05). The BPM had the highest ∑SFA (*p* < 0.01). The TPM had the highest Ca content (*p* < 0.05). Overall, the mineral composition and fatty acid profile of IMC were superior to those of TPM and BPM. The FL exhibited the highest a*_45min_, b*_45min_ (*p* < 0.01), Fe, Zn and C17:1 (*p* < 0.05), and the lowest shear force and drip loss (*p* < 0.01). The HD increased the L*_45min_ compared with the FL (*p* < 0.01). In contrast, the HD showed the highest pH_45min_ and cooking loss (*p* < 0.01). A total of 513 DEGs were identified between FL and HD, which were significantly enriched in pathways such as the cGMP-PKG signaling pathway, glycerophospholipid metabolism, and PPAR signaling pathway. Among these, *SLC16A7*, *GPAM*, *FABP3*, and *TNNC1* were significantly upregulated in the FL group. These genes are proposed as candidate markers potentially involved in de novo lipogenesis, triglyceride synthesis, fatty acid transport, and the regulation of slow oxidative muscle fibers, and may collectively contribute to enhanced intramuscular fat deposition, improved fatty acid composition, and increased tenderness. However, as a correlative transcriptomic study, further functional validation is needed to confirm their causal roles. Our findings provide preliminary molecular insights into the mechanisms underlying meat quality differences in Mongolian horses and lay a foundation for the genetic improvement and conservation of this valuable breed.

## Figures and Tables

**Table 1:** Parameters of primer pairs used for qRT-PCR.

Gene Name	Forward Primer Sequence (5′-3′)	Reverse Primer Sequence (5′-3′)
*GAPDH*	CATCATCCCTGCTTCTACTGG	TCCACGACTGACACGTTAGG
*HOXC10*	GGGACCAACGACTTCGAA	GAGTCTCAGGGAAACTCACTTT
*HOXD8*	TTCAAGCTACCTTCATGTCACT	GAAGTTTACGTTGTTGAGGCAA
*LIMK2*	GAATGGCCTATTTGCATTCCAT	CCTTTTCCTCTCCTCGACTATG
*CRHR2*	TACTCCTACTGCAACACGACTT	GTTGTACTTGATGCCGTTGAAA
*GPAM*	GTTGTTATTCCTGTACTCCCCA	CTTGAACAAACAGCACGTAAGA
*PPM1J*	CATTGTCCGGAATGGTGAAATC	GAAACTCAAGGTGGGTGAATTC

**Table 2:** Chemical and mineral composition of different regions of Mongolian horses (DM).

Items	BPM	TPM	IMC
Chemical composition			
Protein (%)	79.08 ± 1.79	79.26 ± 1.94	81.72 ± 0.68
Fat (%)	12.25 ± 1.94	11.46 ± 1.24	9.95 ± 0.88
Ash (%)	7.17 ± 0.42	7.72 ± 0.27	8.47 ± 0.43
Mineral composition			
Fe (mg/kg)	72.69 ± 2.90 ^b^	71.60 ± 1.45 ^b^	112.43 ± 9.72 ^a^
Zn (mg/kg)	110.45 ± 6.12	93.87 ± 7.01	105.79 ± 6.38
Ca (mg/kg)	457.25 ± 12.27 ^b^	563.83 ± 38.80 ^a^	362.81 ± 24.85 ^c^
Se (μg/kg)	116.15 ± 15.54 ^b^	82.73 ± 32.78 ^b^	231.82 ± 24.79 ^a^

^a,b,c^ Values within a row with different superscripts differ significantly at *p* < 0.05. Results were presented as mean ± SEM. n = 10. BPM: Bulgan province, Mongolia; TPM: Tuv province, Mongolia; IMC: Baotou in Inner Mongolia, China.

**Table 3:** FA composition of different regions of Mongolian horses (%).

Items	BPM	TPM	IMC
C12:0	0.21 ± 0.01	0.17 ± 0.02	0.21 ± 0.02
C14:0	2.98 ± 0.26	2.94 ± 0.31	4.02 ± 0.21
C15:0	0.66 ± 0.03 ^a^	0.57 ± 0.03 ^a^	0.25 ± 0.01 ^b^
C16:0	31.48 ± 0.42 ^a^	30.95 ± 1.28 ^a^	28.14 ± 1.50 ^b^
C17:0	0.46 ± 0.03	0.39 ± 0.03	0.37 ± 0.01
C18:0	7.66 ± 0.61	6.24 ± 0.28	6.29 ± 0.18
C23:0	0.56 ± 0.07	0.80 ± 0.10	0.87 ± 0.18
C14:1	0.37 ± 0.07	0.32 ± 0.05	0.45 ± 0.07
C16:1	6.16 ± 0.74	6.43 ± 0.67	6.99 ± 0.08
C17:1	0.55 ± 0.02 ^b^	0.50 ± 0.03 ^b^	0.71 ± 0.10 ^a^
C18:1n9c	32.61 ± 0.58	32.90 ± 2.39	29.97 ± 1.91
C18:2n6c	7.47 ± 0.76 ^b^	7.81 ± 0.24 ^b^	10.86 ± 1.51 ^a^
C18:3n3	4.03 ± 0.30 ^b^	4.95 ± 0.85 ^b^	6.12 ± 0.98 ^a^
∑SFA ^1^	44.02 ± 0.37 ^a^	42.07 ± 1.54 ^b^	40.14 ± 0.95 ^b^
∑MUFA ^2^	39.69 ± 1.17	40.14 ± 1.86	38.12 ± 1.83
∑PUFA ^3^	11.50 ± 0.96 ^b^	12.75 ± 0.10 ^b^	16.98 ± 2.49 ^a^
PUFA/SFA	0.26 ± 0.02 ^b^	0.31 ± 0.03 ^b^	0.43 ± 0.07 ^a^
∑n-3PUFA ^4^	4.03 ± 0.30 ^b^	4.91 ± 0.85 ^b^	6.12 ± 0.98 ^a^
∑n-6PUFA ^5^	7.47 ± 0.76 ^b^	7.81 ± 0.24 ^b^	10.86 ± 1.51 ^a^
n-6PUFA/n-3 PUFA	1.87 ± 0.15	1.79 ± 0.32	1.79 ± 0.04

^1^ ∑SFA = ∑ (C12:0, C14:0, C15:0, C16:0, C17:0, C18:0, C23:0); ^2^ ∑MUFA = ∑(C14:1, C16:1, C17:1, C18:1n9c); ^3^ ∑PUFA = ∑(C18:2n6c, C18:3n3); ^4^ n-3PUFA: total omega 3 fatty acids, ∑n-3PUFA = ∑(C18:3n3); ^5^ n-6PUFA: total omega 6 fatty acids, ∑n-6PUFA = ∑(C18:2n6c). ^a,b^ Values within a row with different superscripts differ significantly at *p* < 0.05. Results were presented as mean ± SEM. n = 10. BPM: Bulgan province, Mongolia; TPM: Tuv province, Mongolia; IMC: Baotou in Inner Mongolia, China.

**Table 4:** Meat quality indexes of different muscle parts of Mongolian horses.

Items	FL	LT	HD
pH_45min_	6.09 ± 0.04 ^b^	6.11 ± 0.03 ^b^	6.26 ± 0.04 ^a^
L*_45min_	31.13 ± 0.92 ^a^	29.09 ± 0.54 ^ab^	27.74 ± 0.58 ^b^
a*_45min_	14.98 ± 0.58 ^a^	11.01 ± 0.25 ^b^	10.86 ± 0.33 ^b^
b*_45min_	4.73 ± 0.37 ^a^	3.05 ± 0.16 ^b^	2.74 ± 0.25 ^b^
Water loss (%)	13.51 ± 0.78 ^b^	18.89 ± 1.24 ^a^	21.63 ± 1.34 ^a^
Cooking loss (%)	34.41 ± 1.04 ^b^	35.73 ± 1.00 ^b^	40.40 ± 1.78 ^a^
Shear force (N)	75.96 ± 3.58 ^c^	86.87 ± 3.06 ^b^	96.97 ± 2.86 ^a^

^a,b,c^ Values within a row with different superscripts differ significantly at *p* < 0.05. Results were presented as mean ± SEM. n = 20. LT: longissimus thoracis; FL: forelimb; HD: hindlimb.

**Table 5:** Chemical and mineral composition indexes of different muscle parts of Mongolian horses (DM).

Items	FL	LT	HD
Chemical composition			
Protein (%)	79.59 ± 0.85	79.45 ± 0.82	81.85 ± 1.27
Fat (%)	10.93 ± 0.94	13.18 ± 0.95	10.79 ± 1.23
Ash (%)	8.11 ± 0.33	7.37 ± 0.30	7.36 ± 0.34
Mineral composition			
Fe (mg/kg)	95.49 ± 3.44 ^a^	72.06 ± 2.92 ^b^	73.77 ± 4.43 ^b^
Zn (mg/kg)	144.55 ± 10.41 ^a^	103.91 ± 8.66 ^b^	89.12 ± 9.72 ^b^
Ca (mg/kg)	513.09 ± 80.43	538.85 ± 30.33	563.39 ± 23.10
Se (μg/kg)	141.91 ± 14.32	123.79 ± 11.28	138.70 ± 15.30

^a,b^ Values within a row with different superscripts differ significantly at *p* < 0.05. Results were presented as mean ± SEM. n = 20. LT: longissimus thoracis; FL: forelimb; HD: hindlimb.

**Table 6:** FA composition indexes of different muscle parts of Mongolian horses (%).

Items	FL	LT	HD
C12:0	0.24 ± 0.07	0.26 ± 0.06	0.29 ± 0.06
C14:0	3.14 ± 0.24	3.30 ± 0.38	3.79 ± 0.22
C15:0	0.45 ± 0.05	0.53 ± 0.07	0.57 ± 0.07
C16:0	29.27 ± 0.76	29.87 ± 0.75	30.56 ± 0.75
C17:0	0.41 ± 0.03	0.41 ± 0.03	0.48 ± 0.04
C18:0	7.26 ± 0.56	6.91 ± 0.48	6.83 ± 0.42
C23:0	0.82 ± 0.13	0.83 ± 0.10	0.57 ± 0.08
C14:1	0.34 ± 0.05	0.37 ± 0.06	0.42 ± 0.08
C16:1	5.84 ± 0.51	6.22 ± 0.59	6.66 ± 0.53
C17:1	0.73 ± 0.04 ^a^	0.56 ± 0.05 ^b^	0.53 ± 0.05 ^b^
C18:1n9c	31.57 ± 1.56	32.18 ± 1.15	33.39 ± 1.06
C18:2n6c	9.60 ± 1.29	8.80 ± 0.59	7.17 ± 0.70
C18:3n3	5.14 ± 0.52	5.46 ± 0.64	4.35 ± 0.51
∑SFA	41.60 ± 1.03	42.11 ± 0.99	43.09 ± 1.04
∑MUFA	38.31 ± 1.78	39.30 ± 1.22	41.19 ± 1.21
∑PUFA	14.75 ± 1.66	14.27 ± 1.04	11.52 ± 1.17
PUFA/SFA	0.36 ± 0.04	0.34 ± 0.03	0.27 ± 0.03
∑n-3PUFA	5.14 ± 0.52	5.46 ± 0.64	4.35 ± 0.51
∑n-6PUFA	9.60 ± 1.29	8.80 ± 0.59	7.17 ± 0.70
n-6PUFA/n-3 PUFA	1.99 ± 0.25	1.79 ± 0.19	1.73 ± 0.13

^a,b^ Values within a row with different superscripts differ significantly at *p* < 0.05. Results were presented as mean ± SEM. n = 20. LT: longissimus thoracis; FL: forelimb; HD: hindlimb.

**Table 7:** DEGs between the HD and FL (top 10).

DEGs	FL	HD	Up/Down	Log_2_FC	*p*-Value
*TMEM131* ^1^	3.14	0.0 001	up	14.94	<0.01
*TLN1* ^2^	2.34	0.0 001	up	14.51	<0.01
*ABCA5* ^3^	1.34	0.0 001	up	13.71	<0.01
*ZNF865* ^4^	1.41	0.0 001	up	13.54	<0.01
*AKAP6* ^5^	0.0 001	1.23	down	−13.59	0.02
*DDI2* ^6^	0.0 001	1.40	down	−13.77	0.01
*PPP4R3B* ^7^	0.0 001	1.68	down	−14.03	0.01
*TMEM201* ^8^	0.0 001	2.86	down	−14.05	0.02
*DENND1B* ^9^	0.0 001	2.17	down	−14.40	<0.01
*DST* ^10^	0.0 001	5.47	down	−15.74	<0.01

^1^ transmembrane protein 131; ^2^ talin 1; ^3^ ATP binding cassette subfamily A member 5; ^4^ zinc finger protein 865; ^5^ A-kinase anchoring protein 6; ^6^ DNA damage inducible 1 homolog 2; ^7^ protein phosphatase 4 regulatory subunit 3B; ^8^ transmembrane protein 201; ^9^ DENN domain containing 1B; ^10^ dystonin; FL: forelimb; HD: hindlimb.

## Data Availability

The raw sequencing data presented in this study are not readily available due to irreversible hard drive failure and permanent data loss. FastQC for the clean reads used in all downstream analyses are provided in the Supplementary Materials. The derived data supporting the conclusions of this article will be made available by the authors on request.

## Supplementary Materials

The following supporting information can be downloaded at: https://www.mdpi.com/article/10.3390/ani16121788/s1. The quality control reports (FastQC) for the clean reads can be downloaded.
