# Supplementary material for: Meat Quality Characteristics of Mongolian Horses in Inner Mongolia: Regional Superiority and Transcriptomic Insights into Tenderness Differences Between Muscular Locations
Source: Animals (Basel). 2026 Jun 9;16(12):1788. doi: 10.3390/ani16121788 (PMC13295530; doi:10.3390/ani16121788)
Supplement: Supplementary file 1 [file animals-16-01788-s001.zip › QF1 R1.fq_fastqc.zip!/H1_QF_Clean_Data1.fq_fastqc/fastqc_report.html]

H1\_QF\_Clean\_Data1.fq.gz FastQC Report


FastQC Report

Thu 17 Dec 2020  
H1\_QF\_Clean\_Data1.fq.gz

## Summary

- Basic Statistics
- Per base sequence quality
- Per sequence quality scores
- Per base sequence content
- Per base GC content
- Per sequence GC content
- Per base N content
- Sequence Length Distribution
- Sequence Duplication Levels
- Overrepresented sequences
- Kmer Content

## Basic Statistics

| Measure | Value |
| --- | --- |
| Filename | H1\_QF\_Clean\_Data1.fq.gz |
| File type | Conventional base calls |
| Encoding | Sanger / Illumina 1.9 |
| Total Sequences | 26658647 |
| Filtered Sequences | 0 |
| Sequence length | 92-141 |
| %GC | 49 |

## Per base sequence quality

## Per sequence quality scores

## Per base sequence content

## Per base GC content

## Per sequence GC content

## Per base N content

## Sequence Length Distribution

## Sequence Duplication Levels

## Overrepresented sequences

| Sequence | Count | Percentage | Possible Source |
| --- | --- | --- | --- |
| GTTTATTGATAGTTGTGTTGTTGGTGTAAATGAGTGAGGTAGTAGGCCTA | 54903 | 0.20594818634269021 | No Hit |
| GTCAGTATCAAGCGGCTGCTTCGAATCCGAAGTGGTGGCTGGATGTAAAG | 48329 | 0.1812882701811536 | No Hit |
| GGCGATTGTTGATTAGTCGGTTGGGTGAGGGGAATAGGATGCTGGGAAAT | 41662 | 0.15627949910586236 | No Hit |
| GTTTGATATTGGGATAGGGCTGGTGGTTTTATGTTAATGATTGTGGTAAT | 37877 | 0.14208147922885958 | No Hit |
| GTCCTTTGCTGTTATGGATAGCTATTATTTGTTTTGATGTAAGTTGGACT | 34523 | 0.12950019556506376 | No Hit |
| GTTCGATTCCTTCCTTTCTTATTTTAGGTTTACGTAGGTGGGTTCTTCAA | 32602 | 0.12229427847557306 | No Hit |
| GTTGTATATGCGTCTGGATAATCAGAATAGCGTCGTGGCATTCCTGAGAG | 31462 | 0.11801799243600021 | No Hit |
| GCTGGATGTAAAGTGGAATTTTAGTTGGCGTAGGAAACATACAATGAGGA | 30427 | 0.11413557484744068 | No Hit |
| GTGAGATTATACCGAAGCCTGGTAGGATAAGAATATAGACTTCGGGGTGC | 30277 | 0.11357290563170741 | No Hit |
| GTGTAAATGAGTGAGGTAGTAGGCCTAATAAGTTTGTTGAGCCAATGAAT | 27612 | 0.10357614923217971 | No Hit |

## Kmer Content

| Sequence | Count | Obs/Exp Overall | Obs/Exp Max | Max Obs/Exp Position |
| --- | --- | --- | --- | --- |
| CTCCT | 12035865 | 3.9786277 | 10.090176 | 1 |
| CCTCC | 9152935 | 3.6249487 | 4.517507 | 90-94 |
| CTCCA | 8742695 | 3.3904734 | 11.045075 | 1 |
| TCCTC | 10200595 | 3.371953 | 8.383399 | 2 |
| CTTCT | 11548550 | 3.1863835 | 5.7018647 | 6 |
| TCCAG | 8458200 | 2.7358172 | 7.3247156 | 2 |
| TCTTC | 9694435 | 2.6748104 | 5.6868067 | 5 |
| CTTCA | 7859045 | 2.5438962 | 5.99706 | 6 |
| GCTCC | 7350990 | 2.4281828 | 7.3129716 | 1 |
| TCCTT | 8020805 | 2.2130358 | 6.6007285 | 2 |
| TCCAC | 5666340 | 2.1974432 | 6.321095 | 2 |
| TCAGC | 6487185 | 2.0982895 | 5.2049694 | 9 |
| CTCAG | 6380160 | 2.063672 | 5.424328 | 1 |
| GCCAG | 6195765 | 2.002551 | 6.0669465 | 1 |
| GCTGG | 7762545 | 1.7837247 | 7.6925635 | 1 |
| GGCAG | 6517270 | 1.7569058 | 6.949736 | 1 |
| GTTGA | 7050405 | 1.5875659 | 7.887781 | 1 |
| GGGGA | 6490585 | 1.459354 | 5.957765 | 1 |
| GGGAA | 5457535 | 1.440631 | 5.303134 | 1 |
| GTCCT | 5164770 | 1.4239708 | 8.808242 | 1 |
| GGGGG | 7239460 | 1.3864477 | 6.0554156 | 1 |
| TTTTT | 7203810 | 1.3847195 | 5.696952 | 2 |
| GTCCA | 4181335 | 1.3524588 | 9.264929 | 1 |
| GTGGG | 6923740 | 1.326962 | 7.9681964 | 1 |
| GTCAG | 4742295 | 1.279357 | 8.831472 | 1 |
| GTTGG | 6238980 | 1.1966078 | 8.071983 | 1 |
| GTGGA | 5231930 | 1.1772242 | 6.803011 | 1 |
| GTCCC | 3502095 | 1.1568139 | 5.373105 | 1 |
| GTCTG | 4776135 | 1.0982995 | 5.4959455 | 1 |
| GTGCT | 4755115 | 1.0934658 | 6.017671 | 1 |
| GTTTG | 5456095 | 1.0472264 | 5.9247437 | 1 |

Produced by FastQC (version 0.10.1)
