# Supplementary material for: Meat Quality Characteristics of Mongolian Horses in Inner Mongolia: Regional Superiority and Transcriptomic Insights into Tenderness Differences Between Muscular Locations
Source: Animals (Basel). 2026 Jun 9;16(12):1788. doi: 10.3390/ani16121788 (PMC13295530; doi:10.3390/ani16121788)
Supplement: Supplementary file 1 [file animals-16-01788-s001.zip › QF1 R2.fq_fastqc.zip!/H1_QF_Clean_Data2.fq_fastqc/fastqc_report.html]

H1\_QF\_Clean\_Data2.fq.gz FastQC Report


FastQC Report

Thu 17 Dec 2020  
H1\_QF\_Clean\_Data2.fq.gz

## Summary

- Basic Statistics
- Per base sequence quality
- Per sequence quality scores
- Per base sequence content
- Per base GC content
- Per sequence GC content
- Per base N content
- Sequence Length Distribution
- Sequence Duplication Levels
- Overrepresented sequences
- Kmer Content

## Basic Statistics

| Measure | Value |
| --- | --- |
| Filename | H1\_QF\_Clean\_Data2.fq.gz |
| File type | Conventional base calls |
| Encoding | Sanger / Illumina 1.9 |
| Total Sequences | 26658647 |
| Filtered Sequences | 0 |
| Sequence length | 92-141 |
| %GC | 49 |

## Per base sequence quality

## Per sequence quality scores

## Per base sequence content

## Per base GC content

## Per sequence GC content

## Per base N content

## Sequence Length Distribution

## Sequence Duplication Levels

## Overrepresented sequences

| Sequence | Count | Percentage | Possible Source |
| --- | --- | --- | --- |
| CAGGAATCGTCCTAGCTAACTCATCCCTAGATATTGTTCTCCACGATACT | 32665 | 0.12253059954618101 | No Hit |
| CAACAATCGCCTAATCTCAATTCAACAATGGCTAGTCCAACTTACATCAA | 31535 | 0.11829182478765708 | No Hit |

## Kmer Content

| Sequence | Count | Obs/Exp Overall | Obs/Exp Max | Max Obs/Exp Position |
| --- | --- | --- | --- | --- |
| AGGAG | 12097265 | 3.844764 | 4.3691154 | 2 |
| GGAGG | 9092200 | 3.4732342 | 5.812529 | 1 |
| GAGGA | 10193320 | 3.2396505 | 5.736434 | 1 |
| TGGAG | 8536145 | 3.233498 | 5.1161456 | 7 |
| AGAAG | 11730100 | 3.101713 | 4.9449415 | 3 |
| GAAGA | 9855920 | 2.6061356 | 5.9336476 | 2 |
| GGAGA | 8106565 | 2.5764358 | 7.1405582 | 1 |
| GTGGA | 5586945 | 2.116339 | 6.7039585 | 1 |
| GAGAA | 7948285 | 2.1017122 | 5.0199122 | 2 |
| GGAAG | 6065430 | 1.9277204 | 5.9340005 | 1 |
| CTTCA | 6470395 | 1.7485899 | 5.851139 | 1 |
| TTTGA | 4533575 | 1.6886631 | 5.9227366 | 2 |
| GGAAA | 5156500 | 1.3634989 | 6.26785 | 1 |
| GTTTG | 2851295 | 1.2765182 | 7.3848577 | 1 |
| GTTTT | 2435320 | 1.0811517 | 5.819618 | 1 |
| GTTCT | 2633815 | 1.0001624 | 5.2233424 | 1 |
| GTTGA | 2419490 | 0.90882516 | 5.2197785 | 1 |

Produced by FastQC (version 0.10.1)
