# Supplementary material for: Meat Quality Characteristics of Mongolian Horses in Inner Mongolia: Regional Superiority and Transcriptomic Insights into Tenderness Differences Between Muscular Locations
Source: Animals (Basel). 2026 Jun 9;16(12):1788. doi: 10.3390/ani16121788 (PMC13295530; doi:10.3390/ani16121788)
Supplement: Supplementary file 1 [file animals-16-01788-s001.zip › QF2 R1.fq_fastqc.zip!/H6_QF_Clean_Data1.fq_fastqc/fastqc_report.html]

H6\_QF\_Clean\_Data1.fq.gz FastQC Report


FastQC Report

Thu 17 Dec 2020  
H6\_QF\_Clean\_Data1.fq.gz

## Summary

- Basic Statistics
- Per base sequence quality
- Per sequence quality scores
- Per base sequence content
- Per base GC content
- Per sequence GC content
- Per base N content
- Sequence Length Distribution
- Sequence Duplication Levels
- Overrepresented sequences
- Kmer Content

## Basic Statistics

| Measure | Value |
| --- | --- |
| Filename | H6\_QF\_Clean\_Data1.fq.gz |
| File type | Conventional base calls |
| Encoding | Sanger / Illumina 1.9 |
| Total Sequences | 24615473 |
| Filtered Sequences | 0 |
| Sequence length | 92-141 |
| %GC | 51 |

## Per base sequence quality

## Per sequence quality scores

## Per base sequence content

## Per base GC content

## Per sequence GC content

## Per base N content

## Sequence Length Distribution

## Sequence Duplication Levels

## Overrepresented sequences

| Sequence | Count | Percentage | Possible Source |
| --- | --- | --- | --- |
| GTCGGCATGTATTAGCTCTAGAATTACCACAGTTATCCAAGTAGGAGAGG | 65924 | 0.26781528837573015 | No Hit |
| GCTCAATCTCGGGTGGCTGAACGCCACTTGTCCCTCTAAGAAGTTGGGGG | 49325 | 0.20038209300304732 | No Hit |
| GTCAGTATCAAGCGGCTGCTTCGAATCCGAAGTGGTGGCTGGATGTAAAG | 34968 | 0.14205698992662055 | No Hit |
| GTACAAAGGGCAGGGACTTAATCAACGCAAGCTTATGACCCGCACTTACT | 29150 | 0.11842144979298183 | No Hit |
| GCCCCATTGGCTCCTCAGCCAAGCACATACACCAAATGTCTGAACCTGCG | 27451 | 0.11151928707605985 | No Hit |
| GTTTATTGATAGTTGTGTTGTTGGTGTAAATGAGTGAGGTAGTAGGCCTA | 26069 | 0.1059049322351027 | No Hit |

## Kmer Content

| Sequence | Count | Obs/Exp Overall | Obs/Exp Max | Max Obs/Exp Position |
| --- | --- | --- | --- | --- |
| CTCCT | 10440225 | 3.0313563 | 8.706676 | 1 |
| CTCCA | 8489160 | 2.9104395 | 9.585845 | 1 |
| TCCTC | 9634230 | 2.7973328 | 6.9957194 | 2 |
| TCCAG | 7232335 | 2.3109686 | 6.848246 | 2 |
| GCCAG | 6629940 | 2.128301 | 7.1342406 | 1 |
| GCTCC | 7284735 | 2.1249506 | 7.031837 | 1 |
| TCCAC | 5908125 | 2.0255525 | 5.724467 | 2 |
| TCCTT | 7314195 | 1.9701871 | 5.7387123 | 2 |
| GGCAG | 5775460 | 1.7279524 | 6.527022 | 1 |
| GCTGG | 6697675 | 1.6970807 | 7.781016 | 1 |
| GGGAA | 5110155 | 1.6747875 | 5.007547 | 1 |
| GGGGG | 6656285 | 1.5792103 | 6.2827125 | 1 |
| GGGGA | 5515105 | 1.5378736 | 5.600794 | 1 |
| GTTGA | 5301150 | 1.464607 | 7.0709014 | 1 |
| TTTTT | 6140525 | 1.4235543 | 5.3802185 | 2 |
| GTGGG | 5832790 | 1.3774519 | 8.0476265 | 1 |
| GCTCA | 4155855 | 1.3279322 | 6.1576433 | 1 |
| GTCAG | 4391695 | 1.3078846 | 8.252397 | 1 |
| GTCCT | 4580830 | 1.2396326 | 7.8110065 | 1 |
| GTCCA | 3865255 | 1.2350761 | 8.745005 | 1 |
| GTTGG | 4894855 | 1.1506203 | 8.391189 | 1 |
| GTGGT | 4828440 | 1.1350083 | 5.3538785 | 1 |
| GTCTG | 4500160 | 1.1350068 | 6.0022564 | 1 |
| GTGGA | 3867920 | 1.0735849 | 6.0539074 | 1 |
| GTCAA | 2983085 | 1.044147 | 5.370004 | 1 |
| GTCCC | 3372540 | 0.98376685 | 5.2136846 | 1 |
| GTGCT | 3823565 | 0.9643596 | 5.23382 | 1 |
| TCGGC | 3138180 | 0.853168 | 5.02938 | 2 |
| GTCGG | 2849315 | 0.7219696 | 6.9388514 | 1 |

Produced by FastQC (version 0.10.1)
