# Supplementary material for: Meat Quality Characteristics of Mongolian Horses in Inner Mongolia: Regional Superiority and Transcriptomic Insights into Tenderness Differences Between Muscular Locations
Source: Animals (Basel). 2026 Jun 9;16(12):1788. doi: 10.3390/ani16121788 (PMC13295530; doi:10.3390/ani16121788)
Supplement: Supplementary file 1 [file animals-16-01788-s001.zip › QF2 R2.fq_fastqc.zip!/H6_QF_Clean_Data2.fq_fastqc/fastqc_report.html]

H6\_QF\_Clean\_Data2.fq.gz FastQC Report


FastQC Report

Thu 17 Dec 2020  
H6\_QF\_Clean\_Data2.fq.gz

## Summary

- Basic Statistics
- Per base sequence quality
- Per sequence quality scores
- Per base sequence content
- Per base GC content
- Per sequence GC content
- Per base N content
- Sequence Length Distribution
- Sequence Duplication Levels
- Overrepresented sequences
- Kmer Content

## Basic Statistics

| Measure | Value |
| --- | --- |
| Filename | H6\_QF\_Clean\_Data2.fq.gz |
| File type | Conventional base calls |
| Encoding | Sanger / Illumina 1.9 |
| Total Sequences | 24615473 |
| Filtered Sequences | 0 |
| Sequence length | 92-141 |
| %GC | 50 |

## Per base sequence quality

## Per sequence quality scores

## Per base sequence content

## Per base GC content

## Per sequence GC content

## Per base N content

## Sequence Length Distribution

## Sequence Duplication Levels

## Overrepresented sequences

| Sequence | Count | Percentage | Possible Source |
| --- | --- | --- | --- |
| CCTCACCCGGCCCGGACACGGACAGGATTGACAGATTGATAGCTCTTTCT | 70975 | 0.2883349021974918 | No Hit |
| CCTGCCAGTAGCATATGCTTGTCTCAAAGATTAAGCCATGCATGTCTAAG | 41758 | 0.1696412658818297 | No Hit |
| CTCACCCGGCCCGGACACGGACAGGATTGACAGATTGATAGCTCTTTCTC | 34317 | 0.1394123119226675 | No Hit |
| GTTAGTTTTACCCTACTGATGATGTGTTGTTGCCATGGTAATCCTGCTCA | 26377 | 0.1071561777423493 | No Hit |

## Kmer Content

| Sequence | Count | Obs/Exp Overall | Obs/Exp Max | Max Obs/Exp Position |
| --- | --- | --- | --- | --- |
| GAAGA | 9330305 | 2.4296808 | 5.315923 | 2 |
| GGAGA | 7556770 | 2.1222486 | 5.7849646 | 1 |
| TTTTT | 3766600 | 2.045985 | 5.2769556 | 2 |
| GTGGA | 6014835 | 2.0168164 | 5.8477263 | 1 |
| CTTCA | 5022600 | 1.6719178 | 5.3397245 | 1 |
| TTTGA | 3919720 | 1.6108294 | 5.4648876 | 2 |
| GTGAA | 4660115 | 1.4488807 | 6.452928 | 1 |
| GTTTT | 2774580 | 1.361364 | 7.5582285 | 1 |
| TTCAA | 4156580 | 1.354821 | 5.1347556 | 2 |
| GGAAA | 5058200 | 1.3171929 | 5.463129 | 1 |
| TCACC | 4331195 | 1.2332525 | 5.1670403 | 3 |
| GTTTG | 2449730 | 1.0857238 | 6.4039426 | 1 |

Produced by FastQC (version 0.10.1)
