# Supplementary material for: Meat Quality Characteristics of Mongolian Horses in Inner Mongolia: Regional Superiority and Transcriptomic Insights into Tenderness Differences Between Muscular Locations
Source: Animals (Basel). 2026 Jun 9;16(12):1788. doi: 10.3390/ani16121788 (PMC13295530; doi:10.3390/ani16121788)
Supplement: Supplementary file 1 [file animals-16-01788-s001.zip › QF3 R1.fq_fastqc.zip!/H10_QF_Clean_Data1.fq_fastqc/fastqc_report.html]

H10\_QF\_Clean\_Data1.fq.gz FastQC Report


FastQC Report

Thu 17 Dec 2020  
H10\_QF\_Clean\_Data1.fq.gz

## Summary

- Basic Statistics
- Per base sequence quality
- Per sequence quality scores
- Per base sequence content
- Per base GC content
- Per sequence GC content
- Per base N content
- Sequence Length Distribution
- Sequence Duplication Levels
- Overrepresented sequences
- Kmer Content

## Basic Statistics

| Measure | Value |
| --- | --- |
| Filename | H10\_QF\_Clean\_Data1.fq.gz |
| File type | Conventional base calls |
| Encoding | Sanger / Illumina 1.9 |
| Total Sequences | 25940758 |
| Filtered Sequences | 0 |
| Sequence length | 92-141 |
| %GC | 49 |

## Per base sequence quality

## Per sequence quality scores

## Per base sequence content

## Per base GC content

## Per sequence GC content

## Per base N content

## Sequence Length Distribution

## Sequence Duplication Levels

## Overrepresented sequences

| Sequence | Count | Percentage | Possible Source |
| --- | --- | --- | --- |
| GTCAGTATCAAGCGGCTGCTTCGAATCCGAAGTGGTGGCTGGATGTAAAG | 50012 | 0.19279313272187343 | No Hit |
| GTTTATTGATAGTTGTGTTGTTGGTGTAAATGAGTGAGGTAGTAGGCCTA | 47222 | 0.18203785718212243 | No Hit |
| GGCGATTGTTGATTAGTCGGTTGGGTGAGGGGAATAGGATGCTGGGAAAT | 38087 | 0.14682300339874416 | No Hit |
| GTTTGATATTGGGATAGGGCTGGTGGTTTTATGTTAATGATTGTGGTAAT | 37031 | 0.14275218943101048 | No Hit |
| GCTGGATGTAAAGTGGAATTTTAGTTGGCGTAGGAAACATACAATGAGGA | 33456 | 0.12897078797774528 | No Hit |
| GTTCGATTCCTTCCTTTCTTATTTTAGGTTTACGTAGGTGGGTTCTTCAA | 33256 | 0.12819980048385632 | No Hit |
| GTCCTTTGCTGTTATGGATAGCTATTATTTGTTTTGATGTAAGTTGGACT | 32213 | 0.12417910070322541 | No Hit |
| GTGAGATTATACCGAAGCCTGGTAGGATAAGAATATAGACTTCGGGGTGT | 27246 | 0.10503162629249306 | No Hit |
| GTTGTATATGCGTCTGGATAATCAGAATAGCGTCGTGGCATTCCTGAGAG | 27220 | 0.10493139791828751 | No Hit |

## Kmer Content

| Sequence | Count | Obs/Exp Overall | Obs/Exp Max | Max Obs/Exp Position |
| --- | --- | --- | --- | --- |
| CTCCT | 10408705 | 3.6952837 | 8.739864 | 1 |
| CCTCC | 8312560 | 3.6016788 | 4.4558053 | 90-94 |
| CTCCA | 7874370 | 3.2155395 | 9.921792 | 1 |
| TCCTC | 8848015 | 3.1412096 | 7.5203586 | 2 |
| CTTCT | 10249985 | 2.9816403 | 5.340024 | 6 |
| TCTTC | 8740250 | 2.5424697 | 5.2712846 | 7 |
| TCCAG | 7507090 | 2.5300026 | 6.312475 | 2 |
| CTTCA | 7210680 | 2.4126544 | 5.6268 | 6 |
| GCTCC | 6537060 | 2.3375666 | 7.522966 | 1 |
| TCCTT | 7360630 | 2.1411493 | 5.900996 | 2 |
| TCCAC | 5207215 | 2.1263926 | 6.1287827 | 2 |
| GGCTG | 8346295 | 2.032816 | 5.2540965 | 1 |
| GCCAG | 5689290 | 1.9312474 | 6.3382015 | 1 |
| GCCCA | 4615430 | 1.898369 | 5.167695 | 1 |
| GCTGG | 7555675 | 1.8402531 | 8.356121 | 1 |
| GGCAG | 6404490 | 1.7942203 | 7.0826383 | 1 |
| GTTGA | 6649415 | 1.5153923 | 7.2617493 | 1 |
| GGGGA | 6512835 | 1.5058185 | 6.4689636 | 1 |
| TTTTT | 7692330 | 1.5022827 | 6.0512195 | 2 |
| GGGAA | 5430555 | 1.4338483 | 5.2918153 | 1 |
| GGGGG | 6958700 | 1.4088807 | 6.3973536 | 1 |
| GTCCT | 4793010 | 1.404335 | 8.551553 | 1 |
| GTGGG | 6659810 | 1.338682 | 7.514989 | 1 |
| GTCCA | 3940175 | 1.3278984 | 9.183684 | 1 |
| GTCAG | 4468050 | 1.2427355 | 8.808741 | 1 |
| GTCCC | 3435790 | 1.2285933 | 5.8375373 | 1 |
| GTGGA | 5089170 | 1.1682048 | 6.9128265 | 1 |
| GTTGG | 5812450 | 1.1599637 | 7.8835683 | 1 |
| GTCTG | 4611680 | 1.1151491 | 5.3358045 | 1 |
| GTGCT | 4505390 | 1.0894473 | 6.154165 | 1 |
| GTTTG | 5279300 | 1.0459985 | 6.1484776 | 1 |

Produced by FastQC (version 0.10.1)
