# Supplementary material for: Meat Quality Characteristics of Mongolian Horses in Inner Mongolia: Regional Superiority and Transcriptomic Insights into Tenderness Differences Between Muscular Locations
Source: Animals (Basel). 2026 Jun 9;16(12):1788. doi: 10.3390/ani16121788 (PMC13295530; doi:10.3390/ani16121788)
Supplement: Supplementary file 1 [file animals-16-01788-s001.zip › QF3 R2.fq_fastqc.zip!/H10_QF_Clean_Data2.fq_fastqc/fastqc_report.html]

H10\_QF\_Clean\_Data2.fq.gz FastQC Report


FastQC Report

Thu 17 Dec 2020  
H10\_QF\_Clean\_Data2.fq.gz

## Summary

- Basic Statistics
- Per base sequence quality
- Per sequence quality scores
- Per base sequence content
- Per base GC content
- Per sequence GC content
- Per base N content
- Sequence Length Distribution
- Sequence Duplication Levels
- Overrepresented sequences
- Kmer Content

## Basic Statistics

| Measure | Value |
| --- | --- |
| Filename | H10\_QF\_Clean\_Data2.fq.gz |
| File type | Conventional base calls |
| Encoding | Sanger / Illumina 1.9 |
| Total Sequences | 25940758 |
| Filtered Sequences | 0 |
| Sequence length | 92-141 |
| %GC | 49 |

## Per base sequence quality

## Per sequence quality scores

## Per base sequence content

## Per base GC content

## Per sequence GC content

## Per base N content

## Sequence Length Distribution

## Sequence Duplication Levels

## Overrepresented sequences

| Sequence | Count | Percentage | Possible Source |
| --- | --- | --- | --- |
| CAGGAATCGTCCTAGCTAACTCATCCCTAGATATTGTTCTCCACGATACT | 27856 | 0.1073831381488544 | No Hit |
| CAACAATCGCCTAATCTCAATTCAACAATGGCTAGTCCAACTTACATCAA | 26802 | 0.10332003405605958 | No Hit |

## Kmer Content

| Sequence | Count | Obs/Exp Overall | Obs/Exp Max | Max Obs/Exp Position |
| --- | --- | --- | --- | --- |
| AGGAG | 10480660 | 3.5662234 | 4.3592763 | 1 |
| GGAGG | 8301050 | 3.4449902 | 5.89634 | 1 |
| TGGAG | 7746480 | 3.073736 | 4.739094 | 7 |
| GAGGA | 8802425 | 2.9951756 | 5.2637496 | 1 |
| GAAGA | 8841250 | 2.466599 | 5.5987554 | 2 |
| GGAGA | 7169410 | 2.4395144 | 6.8928 | 1 |
| GTGGA | 5165785 | 2.0497386 | 6.938857 | 1 |
| GGAAG | 5739360 | 1.9529151 | 6.0737314 | 1 |
| GGCAG | 5139330 | 1.7945827 | 5.2278337 | 1 |
| GGGAG | 4256320 | 1.7664007 | 5.0006967 | 1 |
| CTTCA | 6170875 | 1.6573719 | 5.092094 | 1 |
| TTTGA | 4559480 | 1.6538334 | 5.5561843 | 2 |
| GGAAA | 5155980 | 1.4384545 | 6.523221 | 1 |
| GTTTG | 2833470 | 1.2535167 | 7.2620955 | 1 |
| GTTTT | 2611370 | 1.1045542 | 6.2374673 | 1 |
| GTTCT | 2640340 | 0.9828185 | 5.0396285 | 1 |
| GTTGA | 2454260 | 0.93108606 | 5.442522 | 1 |

Produced by FastQC (version 0.10.1)
