# Supplementary material for: Meat Quality Characteristics of Mongolian Horses in Inner Mongolia: Regional Superiority and Transcriptomic Insights into Tenderness Differences Between Muscular Locations
Source: Animals (Basel). 2026 Jun 9;16(12):1788. doi: 10.3390/ani16121788 (PMC13295530; doi:10.3390/ani16121788)
Supplement: Supplementary file 1 [file animals-16-01788-s001.zip › QF4 R1.fq_fastqc.zip!/H11_QF_Clean_Data1.fq_fastqc/fastqc_report.html]

H11\_QF\_Clean\_Data1.fq.gz FastQC Report


FastQC Report

Thu 17 Dec 2020  
H11\_QF\_Clean\_Data1.fq.gz

## Summary

- Basic Statistics
- Per base sequence quality
- Per sequence quality scores
- Per base sequence content
- Per base GC content
- Per sequence GC content
- Per base N content
- Sequence Length Distribution
- Sequence Duplication Levels
- Overrepresented sequences
- Kmer Content

## Basic Statistics

| Measure | Value |
| --- | --- |
| Filename | H11\_QF\_Clean\_Data1.fq.gz |
| File type | Conventional base calls |
| Encoding | Sanger / Illumina 1.9 |
| Total Sequences | 25279106 |
| Filtered Sequences | 0 |
| Sequence length | 92-141 |
| %GC | 49 |

## Per base sequence quality

## Per sequence quality scores

## Per base sequence content

## Per base GC content

## Per sequence GC content

## Per base N content

## Sequence Length Distribution

## Sequence Duplication Levels

## Overrepresented sequences

| Sequence | Count | Percentage | Possible Source |
| --- | --- | --- | --- |
| GTTTATTGATAGTTGTGTTGTTGGTGTGAATGAGTGAGGTAGTAGGCCTA | 42812 | 0.16935725495988663 | No Hit |
| GTCAGTATCAAGCGGCTGCTTCGAATCCGAAGTGGTGGCTGGATGTAAAG | 42047 | 0.16633104034612617 | No Hit |
| GTTTCATGTTGTATATGCGTCTGGATAATCAGAATAGCGTCGTGGCATTC | 35635 | 0.14096621929588807 | No Hit |
| GCTGGATGTAAAGTGGAATTTTAGTTGGCGTAGGAAACATACAATGAGGA | 31162 | 0.12327176443660626 | No Hit |
| GGCGATTGTTAATTAGTCGGTTGGGTGAGGGGAATAGGATGCTGGGAAAT | 26363 | 0.10428770700989189 | No Hit |

## Kmer Content

| Sequence | Count | Obs/Exp Overall | Obs/Exp Max | Max Obs/Exp Position |
| --- | --- | --- | --- | --- |
| CTCCT | 10456705 | 3.455102 | 7.9647737 | 1 |
| CCTCC | 8382995 | 3.2144675 | 3.7793255 | 90-94 |
| CTCCA | 8324775 | 3.1707475 | 9.265484 | 1 |
| TCCTC | 9114405 | 3.0115795 | 7.302927 | 2 |
| CTTCT | 10544855 | 3.00236 | 5.368969 | 5 |
| TCTTC | 9121120 | 2.5969903 | 5.2528048 | 7 |
| TCCAG | 7865760 | 2.5923226 | 6.6153874 | 2 |
| CTTCA | 7519155 | 2.467825 | 5.5581465 | 6 |
| TCCTT | 7841520 | 2.232659 | 5.311078 | 2 |
| GCTCC | 6690325 | 2.2198126 | 7.39387 | 1 |
| TCCAC | 5772880 | 2.1987793 | 6.3855457 | 2 |
| GCCAG | 6007795 | 1.9882299 | 6.6930995 | 1 |
| GGCTG | 7949125 | 1.9747262 | 5.1558514 | 1 |
| GGCAG | 6692085 | 1.9163381 | 7.4051785 | 1 |
| GCTGG | 7636195 | 1.8969879 | 8.778733 | 1 |
| GCCCA | 4733315 | 1.8103294 | 5.177119 | 1 |
| GGGGA | 6280020 | 1.5560765 | 6.962271 | 1 |
| TTTTT | 7359215 | 1.5558438 | 6.487579 | 2 |
| GGGGG | 6720030 | 1.4505111 | 6.676237 | 1 |
| GGGAA | 5049435 | 1.4362594 | 5.466509 | 1 |
| GTCCA | 4352350 | 1.4344064 | 9.984452 | 1 |
| GTTGA | 5745610 | 1.4118868 | 6.8329697 | 1 |
| GTCCT | 4812295 | 1.3758702 | 8.031609 | 1 |
| GTGGG | 6380310 | 1.3714769 | 7.696067 | 1 |
| GTCAG | 4516290 | 1.2879218 | 9.360641 | 1 |
| GTGGA | 4775315 | 1.1783352 | 6.4926453 | 1 |
| GTTGG | 5320075 | 1.1388366 | 8.450781 | 1 |
| GTGCT | 4529745 | 1.1206195 | 6.2860937 | 1 |
| GTCTG | 4493285 | 1.1115999 | 5.319922 | 1 |
| GTTTT | 5149640 | 1.0932373 | 5.298839 | 1 |
| GTCCC | 3185740 | 1.0570108 | 5.5276465 | 1 |
| GTTTG | 4834735 | 1.0306549 | 5.7800746 | 1 |
| GTCAC | 2948200 | 0.97163993 | 5.24777 | 1 |

Produced by FastQC (version 0.10.1)
