# Supplementary material for: Meat Quality Characteristics of Mongolian Horses in Inner Mongolia: Regional Superiority and Transcriptomic Insights into Tenderness Differences Between Muscular Locations
Source: Animals (Basel). 2026 Jun 9;16(12):1788. doi: 10.3390/ani16121788 (PMC13295530; doi:10.3390/ani16121788)
Supplement: Supplementary file 1 [file animals-16-01788-s001.zip › QF4 R2.fq_fastqc.zip!/H11_QF_Clean_Data2.fq_fastqc/fastqc_report.html]

H11\_QF\_Clean\_Data2.fq.gz FastQC Report


FastQC Report

Thu 17 Dec 2020  
H11\_QF\_Clean\_Data2.fq.gz

## Summary

- Basic Statistics
- Per base sequence quality
- Per sequence quality scores
- Per base sequence content
- Per base GC content
- Per sequence GC content
- Per base N content
- Sequence Length Distribution
- Sequence Duplication Levels
- Overrepresented sequences
- Kmer Content

## Basic Statistics

| Measure | Value |
| --- | --- |
| Filename | H11\_QF\_Clean\_Data2.fq.gz |
| File type | Conventional base calls |
| Encoding | Sanger / Illumina 1.9 |
| Total Sequences | 25279106 |
| Filtered Sequences | 0 |
| Sequence length | 92-141 |
| %GC | 49 |

## Per base sequence quality

## Per sequence quality scores

## Per base sequence content

## Per base GC content

## Per sequence GC content

## Per base N content

## Sequence Length Distribution

## Sequence Duplication Levels

## Overrepresented sequences

No overrepresented sequences

## Kmer Content

| Sequence | Count | Obs/Exp Overall | Obs/Exp Max | Max Obs/Exp Position |
| --- | --- | --- | --- | --- |
| AGGAG | 10540130 | 3.3301 | 4.0030985 | 1 |
| GGAGG | 8433860 | 3.080347 | 5.428593 | 1 |
| TGGAG | 8205080 | 3.0254183 | 4.5053115 | 7 |
| GAGGA | 9134690 | 2.8860586 | 5.283683 | 1 |
| GAAGA | 9222390 | 2.5205355 | 5.8335366 | 2 |
| GGAGA | 7524475 | 2.377319 | 7.0487256 | 1 |
| TTTTT | 4991030 | 2.2098987 | 5.320059 | 2 |
| GTGGA | 5728580 | 2.1122708 | 7.224631 | 1 |
| GGAAG | 6160685 | 1.9464365 | 6.1451077 | 1 |
| CTTCA | 5945995 | 1.7296621 | 5.197987 | 1 |
| GGCAG | 5277650 | 1.7039865 | 5.173705 | 1 |
| TTTGA | 4439940 | 1.6685474 | 5.645441 | 2 |
| GGAAA | 5274640 | 1.441591 | 6.301584 | 1 |
| GTTTG | 2960395 | 1.286095 | 7.5911508 | 1 |
| GTGAA | 3946740 | 1.258865 | 5.2815056 | 1 |
| GTTTT | 2812280 | 1.233421 | 7.1303744 | 1 |
| GTTCT | 2709865 | 1.0406948 | 5.5189157 | 1 |

Produced by FastQC (version 0.10.1)
