# Supplementary material for: Meat Quality Characteristics of Mongolian Horses in Inner Mongolia: Regional Superiority and Transcriptomic Insights into Tenderness Differences Between Muscular Locations
Source: Animals (Basel). 2026 Jun 9;16(12):1788. doi: 10.3390/ani16121788 (PMC13295530; doi:10.3390/ani16121788)
Supplement: Supplementary file 1 [file animals-16-01788-s001.zip › QF5 R1.fq_fastqc.zip!/H13_QF_Clean_Data1.fq_fastqc/fastqc_report.html]

H13\_QF\_Clean\_Data1.fq.gz FastQC Report


FastQC Report

Thu 17 Dec 2020  
H13\_QF\_Clean\_Data1.fq.gz

## Summary

- Basic Statistics
- Per base sequence quality
- Per sequence quality scores
- Per base sequence content
- Per base GC content
- Per sequence GC content
- Per base N content
- Sequence Length Distribution
- Sequence Duplication Levels
- Overrepresented sequences
- Kmer Content

## Basic Statistics

| Measure | Value |
| --- | --- |
| Filename | H13\_QF\_Clean\_Data1.fq.gz |
| File type | Conventional base calls |
| Encoding | Sanger / Illumina 1.9 |
| Total Sequences | 25022444 |
| Filtered Sequences | 0 |
| Sequence length | 92-141 |
| %GC | 50 |

## Per base sequence quality

## Per sequence quality scores

## Per base sequence content

## Per base GC content

## Per sequence GC content

## Per base N content

## Sequence Length Distribution

## Sequence Duplication Levels

## Overrepresented sequences

| Sequence | Count | Percentage | Possible Source |
| --- | --- | --- | --- |
| GTTTGATATTGGGATAGGGCTGGTGGTTTTATGTTAATGATTGTGGTAAT | 52550 | 0.21001146011157024 | No Hit |
| GCTGGATGTAAAGTGGAATTTTAGTTGGCGTAGGAAACATACAATGAGGA | 35769 | 0.14294766730220276 | No Hit |
| GTTTATTGATAGTTGTGTTGTTGGTGTAAATGAGTGAGGTAGTAGGCCTA | 27363 | 0.10935382650871354 | No Hit |
| GTTCGATTCCTTCCTTTCTTATTTTAGGTTTACGTAGGTGGGTTCTTCAA | 26570 | 0.10618467164918023 | No Hit |

## Kmer Content

| Sequence | Count | Obs/Exp Overall | Obs/Exp Max | Max Obs/Exp Position |
| --- | --- | --- | --- | --- |
| CTCCT | 11524225 | 3.6448064 | 4.750392 | 2 |
| CTCCA | 8863955 | 3.2711468 | 5.8106036 | 6 |
| CCTCC | 8943920 | 3.2125616 | 3.8620732 | 90-94 |
| CTTCT | 11468730 | 3.1938708 | 6.1765075 | 6 |
| TCCTC | 10062990 | 3.1826568 | 5.174268 | 2 |
| TCCAG | 8649775 | 2.8153906 | 5.5917807 | 7 |
| TCTTC | 9700530 | 2.7014534 | 5.5116305 | 5 |
| CTTCA | 7928975 | 2.5764918 | 5.6724353 | 6 |
| GCTCC | 7375160 | 2.336449 | 6.97444 | 1 |
| TCCAC | 5806405 | 2.142791 | 5.6937904 | 2 |
| GCCAG | 6368015 | 2.0761561 | 9.251487 | 1 |
| TTCCT | 7421690 | 2.0668304 | 5.0716305 | 7 |
| GCTGG | 7740715 | 1.9076061 | 10.582269 | 1 |
| GCTTC | 6832075 | 1.9057977 | 6.688011 | 1 |
| GGCAG | 6493790 | 1.8673064 | 5.567922 | 1 |
| GCCCA | 4694740 | 1.7354262 | 6.1267343 | 1 |
| CTGGA | 5998915 | 1.722138 | 5.5157514 | 2 |
| GGAGA | 5461470 | 1.6135254 | 6.0258274 | 1 |
| TTTTT | 7461415 | 1.6110228 | 7.4708858 | 2 |
| TTGAT | 6283980 | 1.5857954 | 5.378582 | 3 |
| GGGAG | 5884525 | 1.4924167 | 5.4174347 | 1 |
| GGGGG | 6844125 | 1.4900782 | 7.045135 | 1 |
| GCCAC | 4027780 | 1.4888822 | 5.7667484 | 1 |
| GCTCA | 4503435 | 1.4658102 | 6.284259 | 1 |
| GGGGA | 5754585 | 1.4594617 | 7.9846396 | 1 |
| GTTGA | 5745030 | 1.4522012 | 7.5490484 | 1 |
| GCTGA | 5018330 | 1.4406366 | 5.947476 | 1 |
| GTCCA | 4345455 | 1.4143896 | 9.989315 | 1 |
| GGGAA | 4682015 | 1.3832449 | 5.5033307 | 1 |
| GTCCT | 4876325 | 1.3602437 | 5.4774055 | 1 |
| GCCTG | 4800665 | 1.341367 | 5.309445 | 1 |
| GTGGG | 6001490 | 1.3044521 | 5.824743 | 1 |
| GTCAG | 4542390 | 1.3040062 | 7.020697 | 1 |
| GCCAT | 3750640 | 1.2207851 | 5.2649417 | 1 |
| TTTGA | 4761120 | 1.2014936 | 5.402715 | 2 |
| GTGGA | 4617010 | 1.1690079 | 6.290714 | 1 |
| GTTGG | 5257105 | 1.1407582 | 7.604699 | 1 |
| GTTCA | 3953395 | 1.1330348 | 6.960006 | 1 |
| GTTTT | 4809365 | 1.0401366 | 6.3147283 | 1 |
| GTTTG | 4664590 | 1.0105044 | 8.796739 | 1 |
| GTTTA | 2751110 | 0.694257 | 5.734978 | 1 |

Produced by FastQC (version 0.10.1)
