# Supplementary material for: Meat Quality Characteristics of Mongolian Horses in Inner Mongolia: Regional Superiority and Transcriptomic Insights into Tenderness Differences Between Muscular Locations
Source: Animals (Basel). 2026 Jun 9;16(12):1788. doi: 10.3390/ani16121788 (PMC13295530; doi:10.3390/ani16121788)
Supplement: Supplementary file 1 [file animals-16-01788-s001.zip › QF5 R2.fq_fastqc.zip!/H13_QF_Clean_Data2.fq_fastqc/fastqc_report.html]

H13\_QF\_Clean\_Data2.fq.gz FastQC Report


FastQC Report

Thu 17 Dec 2020  
H13\_QF\_Clean\_Data2.fq.gz

## Summary

- Basic Statistics
- Per base sequence quality
- Per sequence quality scores
- Per base sequence content
- Per base GC content
- Per sequence GC content
- Per base N content
- Sequence Length Distribution
- Sequence Duplication Levels
- Overrepresented sequences
- Kmer Content

## Basic Statistics

| Measure | Value |
| --- | --- |
| Filename | H13\_QF\_Clean\_Data2.fq.gz |
| File type | Conventional base calls |
| Encoding | Sanger / Illumina 1.9 |
| Total Sequences | 25022444 |
| Filtered Sequences | 0 |
| Sequence length | 92-141 |
| %GC | 50 |

## Per base sequence quality

## Per sequence quality scores

## Per base sequence content

## Per base GC content

## Per sequence GC content

## Per base N content

## Sequence Length Distribution

## Sequence Duplication Levels

## Overrepresented sequences

No overrepresented sequences

## Kmer Content

| Sequence | Count | Obs/Exp Overall | Obs/Exp Max | Max Obs/Exp Position |
| --- | --- | --- | --- | --- |
| AGGAG | 11787230 | 3.5171485 | 4.0214067 | 2 |
| AGAAG | 11600955 | 3.103633 | 5.0098376 | 3 |
| TGGAG | 8775400 | 3.0810502 | 4.672973 | 3 |
| GAGGA | 10194765 | 3.0419788 | 5.998986 | 1 |
| GGAGG | 9095070 | 3.026825 | 5.9384165 | 1 |
| GCTGG | 7870575 | 2.8013139 | 9.2455 | 1 |
| CTGGA | 8545185 | 2.7269332 | 5.1508064 | 2 |
| GGAGA | 8307075 | 2.4787178 | 9.514338 | 1 |
| GCTGA | 6844985 | 2.1843665 | 10.77484 | 1 |
| GAGAA | 7894715 | 2.1120932 | 5.3474793 | 2 |
| GTGGA | 5788585 | 2.032377 | 8.412766 | 1 |
| GGAAG | 6312365 | 1.8835236 | 6.249781 | 1 |
| CTGAA | 6307915 | 1.8048309 | 5.802475 | 2 |
| GCAGA | 6374255 | 1.7287393 | 5.694556 | 1 |
| TTTGA | 4366465 | 1.7063297 | 5.690329 | 2 |
| GGGAG | 4604580 | 1.532397 | 5.7555237 | 1 |
| GCCAG | 5152810 | 1.4166669 | 6.641348 | 1 |
| GCCAA | 5596440 | 1.3795363 | 6.483209 | 1 |
| GGGGA | 4144865 | 1.3794045 | 5.8137984 | 1 |
| GTTTG | 2954220 | 1.2875916 | 9.177978 | 1 |
| GTTTT | 2707235 | 1.2448351 | 5.4580874 | 1 |
| GTGAA | 3926310 | 1.2359877 | 5.054091 | 1 |
| GGGAA | 3864465 | 1.1531036 | 5.1199975 | 1 |
| GTTCT | 2598205 | 1.0292714 | 5.302869 | 1 |
| GTTCA | 2758305 | 0.92863685 | 6.9761243 | 1 |
| GTTGA | 2394245 | 0.8868517 | 6.9483213 | 1 |

Produced by FastQC (version 0.10.1)
