# Supplementary material for: Meat Quality Characteristics of Mongolian Horses in Inner Mongolia: Regional Superiority and Transcriptomic Insights into Tenderness Differences Between Muscular Locations
Source: Animals (Basel). 2026 Jun 9;16(12):1788. doi: 10.3390/ani16121788 (PMC13295530; doi:10.3390/ani16121788)
Supplement: Supplementary file 1 [file animals-16-01788-s001.zip › QF6 R1.fq_fastqc.zip!/H14_QF_Clean_Data1.fq_fastqc/fastqc_report.html]

H14\_QF\_Clean\_Data1.fq.gz FastQC Report


FastQC Report

Thu 17 Dec 2020  
H14\_QF\_Clean\_Data1.fq.gz

## Summary

- Basic Statistics
- Per base sequence quality
- Per sequence quality scores
- Per base sequence content
- Per base GC content
- Per sequence GC content
- Per base N content
- Sequence Length Distribution
- Sequence Duplication Levels
- Overrepresented sequences
- Kmer Content

## Basic Statistics

| Measure | Value |
| --- | --- |
| Filename | H14\_QF\_Clean\_Data1.fq.gz |
| File type | Conventional base calls |
| Encoding | Sanger / Illumina 1.9 |
| Total Sequences | 27002973 |
| Filtered Sequences | 0 |
| Sequence length | 92-141 |
| %GC | 51 |

## Per base sequence quality

## Per sequence quality scores

## Per base sequence content

## Per base GC content

## Per sequence GC content

## Per base N content

## Sequence Length Distribution

## Sequence Duplication Levels

## Overrepresented sequences

| Sequence | Count | Percentage | Possible Source |
| --- | --- | --- | --- |
| GTTTGATATTGGGATAGAGCCGGTGGTTTTATGTTAATGATTGTGGTAAT | 36004 | 0.1333334666519868 | No Hit |

## Kmer Content

| Sequence | Count | Obs/Exp Overall | Obs/Exp Max | Max Obs/Exp Position |
| --- | --- | --- | --- | --- |
| CTCCT | 13469595 | 3.5015366 | 4.8048954 | 1 |
| CTCCA | 10532870 | 3.2363513 | 5.9559627 | 6 |
| CTTCT | 13308380 | 3.216562 | 5.7243185 | 6 |
| TCCTC | 12108155 | 3.1476185 | 5.27001 | 2 |
| CAGCT | 10613100 | 3.048825 | 5.3354964 | 9 |
| TCCAG | 10456125 | 3.0037305 | 6.0732994 | 7 |
| TCTTC | 11481995 | 2.775135 | 5.4540863 | 5 |
| CTTCA | 9117210 | 2.604555 | 5.716694 | 6 |
| GCTCC | 8593545 | 2.2464418 | 6.7966495 | 1 |
| GCCAG | 7213485 | 2.0837903 | 9.249472 | 1 |
| TCCAC | 6683590 | 2.0536137 | 5.2491717 | 2 |
| GGCAG | 7330160 | 1.9797193 | 5.6228695 | 1 |
| GCTGG | 8643815 | 1.9751078 | 10.509204 | 1 |
| GCTTC | 7709935 | 1.8738556 | 6.39035 | 1 |
| CTGGA | 6802300 | 1.8269525 | 5.162966 | 2 |
| GGAGA | 5803805 | 1.7225443 | 6.377084 | 1 |
| TTTTT | 8171960 | 1.707333 | 7.5734754 | 2 |
| GCCCA | 5281915 | 1.6319956 | 5.5826416 | 1 |
| GGGAG | 6370210 | 1.6085153 | 5.514275 | 1 |
| GGGGG | 7110115 | 1.5274322 | 6.956236 | 1 |
| GGGGA | 6036030 | 1.5241328 | 7.9232006 | 1 |
| GTCCA | 5174920 | 1.4865992 | 9.85619 | 1 |
| GCCAC | 4795530 | 1.4817134 | 5.5747147 | 1 |
| GCTCA | 5154645 | 1.4807748 | 6.332035 | 1 |
| GCTGA | 5394745 | 1.4489132 | 5.8413396 | 1 |
| GTCAG | 5224415 | 1.4031665 | 7.076956 | 1 |
| GGGAA | 4712985 | 1.3987936 | 5.3456974 | 1 |
| GCCTG | 5634905 | 1.37718 | 5.3122115 | 1 |
| GTTGA | 5467885 | 1.3653797 | 7.171681 | 1 |
| GTCCT | 5595885 | 1.3600479 | 5.4664955 | 1 |
| GTGGG | 6152395 | 1.3143494 | 5.802363 | 1 |
| GTGGA | 4658800 | 1.169841 | 5.86036 | 1 |
| GTTGG | 5389865 | 1.1450536 | 7.831025 | 1 |
| GTTTT | 5136275 | 1.079093 | 6.487802 | 1 |
| GTTCA | 4027680 | 1.075741 | 5.8601117 | 1 |
| GTTTG | 4497885 | 0.9502495 | 8.290908 | 1 |
| GTTTA | 2784290 | 0.69140077 | 5.9614286 | 1 |

Produced by FastQC (version 0.10.1)
