# Supplementary material for: Meat Quality Characteristics of Mongolian Horses in Inner Mongolia: Regional Superiority and Transcriptomic Insights into Tenderness Differences Between Muscular Locations
Source: Animals (Basel). 2026 Jun 9;16(12):1788. doi: 10.3390/ani16121788 (PMC13295530; doi:10.3390/ani16121788)
Supplement: Supplementary file 1 [file animals-16-01788-s001.zip › QF6 R2.fq_fastqc.zip!/H14_QF_Clean_Data2.fq_fastqc/fastqc_report.html]

H14\_QF\_Clean\_Data2.fq.gz FastQC Report


FastQC Report

Thu 17 Dec 2020  
H14\_QF\_Clean\_Data2.fq.gz

## Summary

- Basic Statistics
- Per base sequence quality
- Per sequence quality scores
- Per base sequence content
- Per base GC content
- Per sequence GC content
- Per base N content
- Sequence Length Distribution
- Sequence Duplication Levels
- Overrepresented sequences
- Kmer Content

## Basic Statistics

| Measure | Value |
| --- | --- |
| Filename | H14\_QF\_Clean\_Data2.fq.gz |
| File type | Conventional base calls |
| Encoding | Sanger / Illumina 1.9 |
| Total Sequences | 27002973 |
| Filtered Sequences | 0 |
| Sequence length | 92-141 |
| %GC | 51 |

## Per base sequence quality

## Per sequence quality scores

## Per base sequence content

## Per base GC content

## Per sequence GC content

## Per base N content

## Sequence Length Distribution

## Sequence Duplication Levels

## Overrepresented sequences

No overrepresented sequences

## Kmer Content

| Sequence | Count | Obs/Exp Overall | Obs/Exp Max | Max Obs/Exp Position |
| --- | --- | --- | --- | --- |
| AGGAG | 13623610 | 3.3644235 | 3.782026 | 2 |
| AGAAG | 13372620 | 3.1322873 | 4.9697485 | 3 |
| TGGAG | 10425835 | 3.0604682 | 4.799365 | 3 |
| GAGGA | 12222575 | 3.0184302 | 5.815637 | 1 |
| AGCTG | 10654925 | 3.0060234 | 4.5257487 | 6 |
| CTGGA | 10354505 | 2.921267 | 5.8851657 | 2 |
| GCTGG | 9597820 | 2.85488 | 9.82737 | 1 |
| GGAGG | 10639155 | 2.770122 | 5.4157386 | 1 |
| TGAAG | 8946940 | 2.491026 | 5.0317874 | 3 |
| GGAGA | 9761990 | 2.4107757 | 8.838835 | 1 |
| GCTGC | 8094890 | 2.314148 | 5.3566403 | 1 |
| GCTGA | 8019380 | 2.2624693 | 10.682946 | 1 |
| GAGAA | 9160650 | 2.1457117 | 5.269417 | 2 |
| GTGGA | 6692050 | 1.9644285 | 8.124558 | 1 |
| CTGAA | 7035770 | 1.8826963 | 5.82312 | 2 |
| GGAAG | 7494525 | 1.8508132 | 5.991207 | 1 |
| GCAGA | 7728680 | 1.8343772 | 5.99318 | 1 |
| TTTGA | 4775075 | 1.7816724 | 5.4333043 | 2 |
| GCCAA | 6435210 | 1.4679488 | 6.8134284 | 1 |
| GCCAG | 6007855 | 1.4449105 | 6.544109 | 1 |
| GGGAG | 5358710 | 1.39525 | 5.1353536 | 1 |
| GTTTT | 3142300 | 1.3936503 | 5.9735117 | 1 |
| GTTTG | 3299200 | 1.2978652 | 8.848368 | 1 |
| GTGAA | 4600665 | 1.2809267 | 5.159609 | 1 |
| GGGGA | 4765770 | 1.240866 | 5.2288737 | 1 |
| GTTCT | 2895550 | 1.0947548 | 5.2959456 | 1 |
| GTTCA | 3160600 | 1.0053024 | 7.541958 | 1 |
| GTTGA | 2517780 | 0.83325917 | 6.2999454 | 1 |
| GTTTA | 2005685 | 0.74835956 | 5.300494 | 1 |

Produced by FastQC (version 0.10.1)
