# Supplementary material for: Meat Quality Characteristics of Mongolian Horses in Inner Mongolia: Regional Superiority and Transcriptomic Insights into Tenderness Differences Between Muscular Locations
Source: Animals (Basel). 2026 Jun 9;16(12):1788. doi: 10.3390/ani16121788 (PMC13295530; doi:10.3390/ani16121788)
Supplement: Supplementary file 1 [file animals-16-01788-s001.zip › QF7 R1.fq_fastqc.zip!/H15_QF_Clean_Data1.fq_fastqc/fastqc_report.html]

H15\_QF\_Clean\_Data1.fq.gz FastQC Report


FastQC Report

Thu 17 Dec 2020  
H15\_QF\_Clean\_Data1.fq.gz

## Summary

- Basic Statistics
- Per base sequence quality
- Per sequence quality scores
- Per base sequence content
- Per base GC content
- Per sequence GC content
- Per base N content
- Sequence Length Distribution
- Sequence Duplication Levels
- Overrepresented sequences
- Kmer Content

## Basic Statistics

| Measure | Value |
| --- | --- |
| Filename | H15\_QF\_Clean\_Data1.fq.gz |
| File type | Conventional base calls |
| Encoding | Sanger / Illumina 1.9 |
| Total Sequences | 25071226 |
| Filtered Sequences | 0 |
| Sequence length | 92-141 |
| %GC | 51 |

## Per base sequence quality

## Per sequence quality scores

## Per base sequence content

## Per base GC content

## Per sequence GC content

## Per base N content

## Sequence Length Distribution

## Sequence Duplication Levels

## Overrepresented sequences

| Sequence | Count | Percentage | Possible Source |
| --- | --- | --- | --- |
| GTTTGATATTGGGATAGAGCTGGTGGTTTTATGTTAATGATTGTGGTAAT | 30297 | 0.12084371143238068 | No Hit |

## Kmer Content

| Sequence | Count | Obs/Exp Overall | Obs/Exp Max | Max Obs/Exp Position |
| --- | --- | --- | --- | --- |
| CTCCT | 12420605 | 3.4411476 | 4.523714 | 1 |
| CTTCT | 12698265 | 3.2655294 | 5.7469187 | 6 |
| CTCCA | 9749085 | 3.2125003 | 5.735045 | 6 |
| TCCTC | 11242400 | 3.1147242 | 5.3417463 | 2 |
| TCCAG | 9549240 | 2.9401083 | 5.886592 | 7 |
| CAGCT | 9473375 | 2.9167504 | 5.239932 | 9 |
| TCTTC | 10932995 | 2.8115664 | 5.4252896 | 5 |
| CTTCA | 8872640 | 2.7138188 | 5.801164 | 6 |
| GCTCC | 7983705 | 2.2265453 | 6.7111807 | 1 |
| GCCAG | 6975190 | 2.1618078 | 9.173767 | 1 |
| TCCAC | 6465135 | 2.1303792 | 5.5760546 | 2 |
| GGCAG | 6892170 | 1.9958698 | 6.213432 | 1 |
| GCTGG | 8121130 | 1.977306 | 9.642423 | 1 |
| GCTTC | 7426195 | 1.9223925 | 6.338335 | 1 |
| GGAGA | 5298795 | 1.6940287 | 5.9538264 | 1 |
| GCCCA | 5027465 | 1.6676116 | 5.511462 | 1 |
| TTTTT | 7315690 | 1.6209209 | 7.279297 | 2 |
| GGGAG | 5773390 | 1.5621485 | 5.441299 | 1 |
| GGGGG | 6637750 | 1.5200576 | 7.187245 | 1 |
| GGGGA | 5544945 | 1.5003365 | 7.7904925 | 1 |
| GCCAC | 4463675 | 1.4806023 | 5.5206914 | 1 |
| GTCCA | 4762430 | 1.466301 | 10.378112 | 1 |
| GCTGA | 4898200 | 1.4091142 | 5.27375 | 1 |
| GGGAA | 4348795 | 1.3903129 | 5.232459 | 1 |
| GTCAG | 4804415 | 1.3821343 | 7.5928025 | 1 |
| GTTGA | 5160750 | 1.3780696 | 8.308603 | 1 |
| GCTCA | 4470335 | 1.3763679 | 5.639419 | 1 |
| GTCCT | 5245860 | 1.3579772 | 6.2570004 | 1 |
| GTGGG | 5777310 | 1.3143116 | 6.6547437 | 1 |
| GTTCT | 4923575 | 1.183055 | 5.181532 | 1 |
| GTTGG | 5150660 | 1.164043 | 8.273596 | 1 |
| GTGGA | 4298465 | 1.1554159 | 6.55705 | 1 |
| GTTCA | 4005775 | 1.1448003 | 6.482007 | 1 |
| GTTTT | 4677800 | 1.0433133 | 6.633695 | 1 |
| GTTTG | 4093360 | 0.9190085 | 7.7401586 | 1 |
| GTTTA | 2520455 | 0.6686068 | 5.662965 | 1 |

Produced by FastQC (version 0.10.1)
