# Supplementary material for: Meat Quality Characteristics of Mongolian Horses in Inner Mongolia: Regional Superiority and Transcriptomic Insights into Tenderness Differences Between Muscular Locations
Source: Animals (Basel). 2026 Jun 9;16(12):1788. doi: 10.3390/ani16121788 (PMC13295530; doi:10.3390/ani16121788)
Supplement: Supplementary file 1 [file animals-16-01788-s001.zip › QF7 R2.fq_fastqc.zip!/H15_QF_Clean_Data2.fq_fastqc/fastqc_report.html]

H15\_QF\_Clean\_Data2.fq.gz FastQC Report


FastQC Report

Thu 17 Dec 2020  
H15\_QF\_Clean\_Data2.fq.gz

## Summary

- Basic Statistics
- Per base sequence quality
- Per sequence quality scores
- Per base sequence content
- Per base GC content
- Per sequence GC content
- Per base N content
- Sequence Length Distribution
- Sequence Duplication Levels
- Overrepresented sequences
- Kmer Content

## Basic Statistics

| Measure | Value |
| --- | --- |
| Filename | H15\_QF\_Clean\_Data2.fq.gz |
| File type | Conventional base calls |
| Encoding | Sanger / Illumina 1.9 |
| Total Sequences | 25071226 |
| Filtered Sequences | 0 |
| Sequence length | 92-141 |
| %GC | 51 |

## Per base sequence quality

## Per sequence quality scores

## Per base sequence content

## Per base GC content

## Per sequence GC content

## Per base N content

## Sequence Length Distribution

## Sequence Duplication Levels

## Overrepresented sequences

No overrepresented sequences

## Kmer Content

| Sequence | Count | Obs/Exp Overall | Obs/Exp Max | Max Obs/Exp Position |
| --- | --- | --- | --- | --- |
| AGGAG | 12661390 | 3.3259473 | 4.0371885 | 2 |
| AGAAG | 12776550 | 3.1903584 | 5.0956388 | 3 |
| TGGAG | 9672860 | 3.0303502 | 4.525098 | 3 |
| GAGGA | 11390930 | 2.9922175 | 5.9705367 | 1 |
| CTGGA | 9411800 | 2.8397505 | 5.2640796 | 2 |
| GCTGG | 8627085 | 2.7382915 | 8.625126 | 1 |
| GGAGG | 9804260 | 2.7092993 | 5.224953 | 1 |
| TGAAG | 8710720 | 2.5940828 | 5.072641 | 3 |
| GGAGA | 9087670 | 2.3871875 | 8.668633 | 1 |
| GCTGA | 7624135 | 2.300372 | 10.057225 | 1 |
| GAGAA | 8561655 | 2.1378813 | 5.2877855 | 2 |
| GTGGA | 6523990 | 2.0438604 | 8.560295 | 1 |
| CTGAA | 6807190 | 1.952393 | 5.6063256 | 2 |
| GGAAG | 6989965 | 1.8361535 | 5.906008 | 1 |
| GCAGA | 7209895 | 1.8240321 | 5.7956944 | 1 |
| TTTGA | 4430695 | 1.7840264 | 5.6891565 | 2 |
| GCCAA | 6052860 | 1.4748021 | 6.6548853 | 1 |
| GCCAG | 5715000 | 1.4648646 | 6.5191836 | 1 |
| GGAAA | 5668320 | 1.4154032 | 5.1095557 | 1 |
| GTTTT | 2878850 | 1.3824594 | 6.484507 | 1 |
| GTGAA | 4405230 | 1.3118929 | 5.5839143 | 1 |
| GTTTG | 3085450 | 1.3069413 | 9.082586 | 1 |
| GTTCT | 2752515 | 1.122889 | 6.1504226 | 1 |
| GTTCA | 3034235 | 1.0378927 | 7.4065366 | 1 |
| GTTGA | 2391815 | 0.8494958 | 6.5799804 | 1 |
| GTTTA | 1832350 | 0.73779863 | 5.1174827 | 1 |

Produced by FastQC (version 0.10.1)
