# Supplementary material for: Meat Quality Characteristics of Mongolian Horses in Inner Mongolia: Regional Superiority and Transcriptomic Insights into Tenderness Differences Between Muscular Locations
Source: Animals (Basel). 2026 Jun 9;16(12):1788. doi: 10.3390/ani16121788 (PMC13295530; doi:10.3390/ani16121788)
Supplement: Supplementary file 1 [file animals-16-01788-s001.zip › QF8 R1.fq_fastqc.zip!/H18_QF_Clean_Data1.fq_fastqc/fastqc_report.html]

H18\_QF\_Clean\_Data1.fq.gz FastQC Report


FastQC Report

Thu 17 Dec 2020  
H18\_QF\_Clean\_Data1.fq.gz

## Summary

- Basic Statistics
- Per base sequence quality
- Per sequence quality scores
- Per base sequence content
- Per base GC content
- Per sequence GC content
- Per base N content
- Sequence Length Distribution
- Sequence Duplication Levels
- Overrepresented sequences
- Kmer Content

## Basic Statistics

| Measure | Value |
| --- | --- |
| Filename | H18\_QF\_Clean\_Data1.fq.gz |
| File type | Conventional base calls |
| Encoding | Sanger / Illumina 1.9 |
| Total Sequences | 26088230 |
| Filtered Sequences | 0 |
| Sequence length | 92-141 |
| %GC | 50 |

## Per base sequence quality

## Per sequence quality scores

## Per base sequence content

## Per base GC content

## Per sequence GC content

## Per base N content

## Sequence Length Distribution

## Sequence Duplication Levels

## Overrepresented sequences

| Sequence | Count | Percentage | Possible Source |
| --- | --- | --- | --- |
| GTTTCATGTTGTATATGCGTCTGGATAATCAGAATAGCGTCGTGGCATTC | 47067 | 0.18041469275608196 | No Hit |
| GTTTGATATTGGGATAGAGCTGGTGGTTTTATGTTAATGATTGTGGTAAT | 35973 | 0.13788976868112554 | No Hit |
| GTCAGTATCAAGCGGCTGCTTCGAATCCGAAGTGGTGGCTGGATGTAAAG | 34526 | 0.1323432061124883 | No Hit |
| GTTTATTGATAGTTGTGTTGTTGGTGTGAATGAGTGAGGTAGTAGGCCTA | 34453 | 0.13206338643901866 | No Hit |
| GCTGGATGTAAAGTGGAATTTTAGTTGGCGTAGGAAACATACAATGAGGA | 32623 | 0.12504872887121893 | No Hit |

## Kmer Content

| Sequence | Count | Obs/Exp Overall | Obs/Exp Max | Max Obs/Exp Position |
| --- | --- | --- | --- | --- |
| CTCCT | 12377905 | 3.587793 | 5.1874394 | 1 |
| CTCCA | 9555900 | 3.2699134 | 6.313205 | 1 |
| CTTCT | 12324385 | 3.2189248 | 5.9771843 | 6 |
| TCCTC | 11000780 | 3.188627 | 6.751644 | 2 |
| CCTCC | 9653410 | 3.1052437 | 3.692356 | 35-39 |
| TCCAG | 9265025 | 2.8337333 | 6.7941837 | 2 |
| TCTTC | 10562580 | 2.7587707 | 5.6502767 | 5 |
| CTTCA | 8634360 | 2.6623197 | 5.934624 | 6 |
| GCTCC | 7861755 | 2.2603834 | 7.386018 | 1 |
| TCCAC | 6474690 | 2.2155607 | 6.585168 | 2 |
| GCCAG | 6926620 | 2.101436 | 8.258704 | 1 |
| GCTGG | 8221350 | 1.8884312 | 9.304001 | 1 |
| GGCAG | 6963775 | 1.8883706 | 6.522423 | 1 |
| GCTTC | 7247905 | 1.8777616 | 5.7472215 | 1 |
| GCCCA | 5229540 | 1.77505 | 5.4355345 | 1 |
| GCCAC | 4606130 | 1.5634474 | 5.30799 | 1 |
| GTCCA | 4942415 | 1.5116513 | 12.172027 | 1 |
| GGGGG | 7238485 | 1.47413 | 6.1877613 | 1 |
| GGGAG | 6080465 | 1.4737608 | 5.0932064 | 1 |
| TTTTT | 6945105 | 1.4728382 | 6.05237 | 2 |
| GGGGA | 6040565 | 1.46409 | 7.07165 | 1 |
| GTTGA | 5880700 | 1.4486197 | 9.10666 | 1 |
| GCTCA | 4666515 | 1.4272665 | 5.335468 | 1 |
| GGGAA | 4892545 | 1.4113253 | 5.7934294 | 1 |
| GTCCT | 5282470 | 1.3685635 | 8.226173 | 1 |
| GTCAG | 4942155 | 1.3510658 | 9.951697 | 1 |
| GTGGG | 6481380 | 1.3306787 | 7.6945796 | 1 |
| GTGGA | 4883610 | 1.1932976 | 7.329141 | 1 |
| GTTCT | 4954495 | 1.1566252 | 5.0211143 | 1 |
| GTCAT | 4195520 | 1.1562814 | 5.673063 | 1 |
| GTTGG | 5525235 | 1.1435997 | 9.676335 | 1 |
| GTCTG | 4874340 | 1.1287336 | 5.5685797 | 1 |
| GTGCT | 4844870 | 1.1219094 | 5.8779798 | 1 |
| GTTCA | 4036030 | 1.112326 | 5.6648674 | 1 |
| GTTTT | 4918320 | 1.0346069 | 6.2258935 | 1 |
| GTCAA | 3132570 | 1.0192083 | 5.273563 | 1 |
| GTTTG | 4834700 | 1.0088125 | 7.4937806 | 1 |
| GTCAC | 3207345 | 0.9809754 | 6.1509194 | 1 |
| GTTTC | 4030910 | 0.9410146 | 5.4142838 | 1 |
| GTGCA | 3219755 | 0.88020307 | 5.3485007 | 1 |
| GTTTA | 2863390 | 0.7110883 | 5.961485 | 1 |

Produced by FastQC (version 0.10.1)
