# Supplementary material for: Meat Quality Characteristics of Mongolian Horses in Inner Mongolia: Regional Superiority and Transcriptomic Insights into Tenderness Differences Between Muscular Locations
Source: Animals (Basel). 2026 Jun 9;16(12):1788. doi: 10.3390/ani16121788 (PMC13295530; doi:10.3390/ani16121788)
Supplement: Supplementary file 1 [file animals-16-01788-s001.zip › QF8 R2.fq_fastqc.zip!/H18_QF_Clean_Data2.fq_fastqc/fastqc_report.html]

H18\_QF\_Clean\_Data2.fq.gz FastQC Report


FastQC Report

Thu 17 Dec 2020  
H18\_QF\_Clean\_Data2.fq.gz

## Summary

- Basic Statistics
- Per base sequence quality
- Per sequence quality scores
- Per base sequence content
- Per base GC content
- Per sequence GC content
- Per base N content
- Sequence Length Distribution
- Sequence Duplication Levels
- Overrepresented sequences
- Kmer Content

## Basic Statistics

| Measure | Value |
| --- | --- |
| Filename | H18\_QF\_Clean\_Data2.fq.gz |
| File type | Conventional base calls |
| Encoding | Sanger / Illumina 1.9 |
| Total Sequences | 26088230 |
| Filtered Sequences | 0 |
| Sequence length | 92-141 |
| %GC | 50 |

## Per base sequence quality

## Per sequence quality scores

## Per base sequence content

## Per base GC content

## Per sequence GC content

## Per base N content

## Sequence Length Distribution

## Sequence Duplication Levels

## Overrepresented sequences

No overrepresented sequences

## Kmer Content

| Sequence | Count | Obs/Exp Overall | Obs/Exp Max | Max Obs/Exp Position |
| --- | --- | --- | --- | --- |
| AGGAG | 12645630 | 3.4623752 | 4.4097147 | 2 |
| AGAAG | 12510950 | 3.133711 | 5.2323656 | 3 |
| TGGAG | 9431585 | 3.0732758 | 4.3570113 | 7 |
| GAGGA | 11146485 | 3.0519092 | 6.3154416 | 1 |
| GGAGG | 9787445 | 2.9293284 | 5.724254 | 1 |
| GCTGG | 8422010 | 2.7651894 | 6.413141 | 1 |
| GAAGA | 10751610 | 2.693036 | 5.9945974 | 2 |
| GGAGA | 8962130 | 2.4538329 | 8.978364 | 1 |
| TTTTT | 4721290 | 2.362736 | 5.029679 | 2 |
| GCTGA | 7263425 | 2.1816523 | 7.1806216 | 1 |
| GAGAA | 8577600 | 2.1484954 | 5.80789 | 2 |
| GTGGA | 6464255 | 2.1063733 | 8.577448 | 1 |
| CCAGC | 8130305 | 1.9058437 | 5.205236 | 8 |
| GGAAG | 6943585 | 1.9011549 | 6.7471423 | 1 |
| TTTGA | 4520410 | 1.7459468 | 5.956157 | 2 |
| GCCAG | 5522870 | 1.404488 | 5.127243 | 1 |
| GCCAA | 5943340 | 1.3826706 | 5.0969243 | 1 |
| GGAAA | 5494740 | 1.3763086 | 6.260944 | 1 |
| GTTTG | 3079255 | 1.3000615 | 8.797101 | 1 |
| GTGAA | 4223290 | 1.2589334 | 5.9323487 | 1 |
| GTTTT | 2702380 | 1.2421751 | 6.9548125 | 1 |
| GTTCT | 2713785 | 1.0561385 | 5.928603 | 1 |
| GTTCA | 2945505 | 0.9632124 | 6.199988 | 1 |
| GTTGA | 2412475 | 0.85585046 | 6.535019 | 1 |

Produced by FastQC (version 0.10.1)
