# Supplementary material for: Meat Quality Characteristics of Mongolian Horses in Inner Mongolia: Regional Superiority and Transcriptomic Insights into Tenderness Differences Between Muscular Locations
Source: Animals (Basel). 2026 Jun 9;16(12):1788. doi: 10.3390/ani16121788 (PMC13295530; doi:10.3390/ani16121788)
Supplement: Supplementary file 1 [file animals-16-01788-s001.zip › TB1 R1.fq_fastqc.zip!/H1_TB_Clean_Data1.fq_fastqc/fastqc_report.html]

H1\_TB\_Clean\_Data1.fq.gz FastQC Report


FastQC Report

Thu 17 Dec 2020  
H1\_TB\_Clean\_Data1.fq.gz

## Summary

- Basic Statistics
- Per base sequence quality
- Per sequence quality scores
- Per base sequence content
- Per base GC content
- Per sequence GC content
- Per base N content
- Sequence Length Distribution
- Sequence Duplication Levels
- Overrepresented sequences
- Kmer Content

## Basic Statistics

| Measure | Value |
| --- | --- |
| Filename | H1\_TB\_Clean\_Data1.fq.gz |
| File type | Conventional base calls |
| Encoding | Sanger / Illumina 1.9 |
| Total Sequences | 26410034 |
| Filtered Sequences | 0 |
| Sequence length | 92-141 |
| %GC | 50 |

## Per base sequence quality

## Per sequence quality scores

## Per base sequence content

## Per base GC content

## Per sequence GC content

## Per base N content

## Sequence Length Distribution

## Sequence Duplication Levels

## Overrepresented sequences

| Sequence | Count | Percentage | Possible Source |
| --- | --- | --- | --- |
| GTTTATTGATAGTTGTGTTGTTGGTGTAAATGAGTGAGGTAGTAGGCCTA | 51169 | 0.19374833065341757 | No Hit |
| GTCAGTATCAAGCGGCTGCTTCGAATCCGAAGTGGTGGCTGGATGTAAAG | 49121 | 0.18599370224210995 | No Hit |
| GGCGATTGTTGATTAGTCGGTTGGGTGAGGGGAATAGGATGCTGGGAAAT | 40788 | 0.15444130060567132 | No Hit |
| GTTTGATATTGGGATAGGGCTGGTGGTTTTATGTTAATGATTGTGGTAAT | 35782 | 0.13548638369795357 | No Hit |
| GTCCTTTGCTGTTATGGATAGCTATTATTTGTTTTGATGTAAGTTGGACT | 35222 | 0.13336597749173668 | No Hit |
| GTTCGATTCCTTCCTTTCTTATTTTAGGTTTACGTAGGTGGGTTCTTCAA | 34198 | 0.12948866328608286 | No Hit |
| GCTGGATGTAAAGTGGAATTTTAGTTGGCGTAGGAAACATACAATGAGGA | 29641 | 0.11223385778299264 | No Hit |
| GTTGTATATGCGTCTGGATAATCAGAATAGCGTCGTGGCATTCCTGAGAG | 29571 | 0.11196880700721551 | No Hit |
| GTGAGATTATACCGAAGCCTGGTAGGATAAGAATATAGACTTCGGGGTGC | 29054 | 0.11001121770611882 | No Hit |

## Kmer Content

| Sequence | Count | Obs/Exp Overall | Obs/Exp Max | Max Obs/Exp Position |
| --- | --- | --- | --- | --- |
| CTCCT | 12553680 | 4.113072 | 11.02548 | 1 |
| CCTCC | 9438740 | 3.6552794 | 4.570802 | 3 |
| TCCTC | 10666195 | 3.4946587 | 8.8866205 | 2 |
| CTCCA | 9035855 | 3.4798024 | 11.670085 | 1 |
| CTTCT | 11737885 | 3.2536716 | 6.009302 | 6 |
| TCCAG | 8800170 | 2.8489337 | 7.676425 | 2 |
| TCTTC | 9804935 | 2.7178698 | 5.798192 | 5 |
| TCTCC | 7963860 | 2.6092687 | 5.002575 | 7 |
| CTTCA | 7891630 | 2.5712287 | 5.8911166 | 6 |
| GCTCC | 7518275 | 2.447545 | 7.4402514 | 1 |
| TCCTT | 7972500 | 2.2099297 | 6.67248 | 2 |
| TCCAC | 5666110 | 2.1820784 | 6.2435136 | 2 |
| TCAGC | 6597960 | 2.1359985 | 5.04669 | 9 |
| CTCAG | 6288410 | 2.035786 | 5.731501 | 1 |
| GCCAG | 6165885 | 1.9833709 | 6.0444117 | 1 |
| GCTGG | 7663320 | 1.7629554 | 7.1837997 | 1 |
| GGCAG | 6357980 | 1.719229 | 6.7875953 | 1 |
| GTTGA | 6889125 | 1.5861709 | 7.7031426 | 1 |
| GGGGA | 6482120 | 1.4734572 | 5.7676554 | 1 |
| GGGAA | 5327030 | 1.4324466 | 5.1965566 | 1 |
| GTCCT | 5164125 | 1.422322 | 8.611078 | 1 |
| TTTTT | 7000190 | 1.3889003 | 5.7724485 | 2 |
| GTCCA | 4262100 | 1.3797963 | 9.104137 | 1 |
| GGGGG | 7161200 | 1.3760474 | 5.656594 | 1 |
| GTGGG | 6894365 | 1.3332899 | 7.3935413 | 1 |
| GTCAG | 4794580 | 1.3048117 | 9.066282 | 1 |
| GTTGG | 6157705 | 1.1984831 | 7.5551515 | 1 |
| GTGGA | 5129935 | 1.1735864 | 6.5982056 | 1 |
| GTCCC | 3514635 | 1.1441758 | 5.189137 | 1 |
| GTGCT | 4771045 | 1.1046395 | 6.036573 | 1 |
| GTCTG | 4685410 | 1.0848126 | 5.3430877 | 1 |
| GTTTG | 5253755 | 1.029119 | 5.785646 | 1 |

Produced by FastQC (version 0.10.1)
