# Supplementary material for: Meat Quality Characteristics of Mongolian Horses in Inner Mongolia: Regional Superiority and Transcriptomic Insights into Tenderness Differences Between Muscular Locations
Source: Animals (Basel). 2026 Jun 9;16(12):1788. doi: 10.3390/ani16121788 (PMC13295530; doi:10.3390/ani16121788)
Supplement: Supplementary file 1 [file animals-16-01788-s001.zip › TB1 R2.fq_fastqc.zip!/H1_TB_Clean_Data2.fq_fastqc/fastqc_report.html]

H1\_TB\_Clean\_Data2.fq.gz FastQC Report


FastQC Report

Thu 17 Dec 2020  
H1\_TB\_Clean\_Data2.fq.gz

## Summary

- Basic Statistics
- Per base sequence quality
- Per sequence quality scores
- Per base sequence content
- Per base GC content
- Per sequence GC content
- Per base N content
- Sequence Length Distribution
- Sequence Duplication Levels
- Overrepresented sequences
- Kmer Content

## Basic Statistics

| Measure | Value |
| --- | --- |
| Filename | H1\_TB\_Clean\_Data2.fq.gz |
| File type | Conventional base calls |
| Encoding | Sanger / Illumina 1.9 |
| Total Sequences | 26410034 |
| Filtered Sequences | 0 |
| Sequence length | 92-141 |
| %GC | 49 |

## Per base sequence quality

## Per sequence quality scores

## Per base sequence content

## Per base GC content

## Per sequence GC content

## Per base N content

## Sequence Length Distribution

## Sequence Duplication Levels

## Overrepresented sequences

| Sequence | Count | Percentage | Possible Source |
| --- | --- | --- | --- |
| CAGGAATCGTCCTAGCTAACTCATCCCTAGATATTGTTCTCCACGATACT | 33699 | 0.1275992298987574 | No Hit |
| CAACAATCGCCTAATCTCAATTCAACAATGGCTAGTCCAACTTACATCAA | 31816 | 0.120469364030353 | No Hit |

## Kmer Content

| Sequence | Count | Obs/Exp Overall | Obs/Exp Max | Max Obs/Exp Position |
| --- | --- | --- | --- | --- |
| AGGAG | 12563605 | 3.9680855 | 4.4638605 | 2 |
| GGAGG | 9366480 | 3.5140526 | 5.7101088 | 1 |
| GAGGA | 10632555 | 3.3581834 | 5.801801 | 1 |
| TGGAG | 8792430 | 3.3199198 | 5.082368 | 7 |
| AGAAG | 11919115 | 3.1691709 | 5.15214 | 3 |
| GAAGA | 10023310 | 2.6650958 | 6.027205 | 2 |
| GGAGA | 8360785 | 2.6406682 | 6.9846215 | 1 |
| GAGAA | 8042065 | 2.1383028 | 5.043817 | 2 |
| GTGGA | 5570555 | 2.103377 | 6.4766126 | 1 |
| GGAAG | 6085285 | 1.9219748 | 5.7797613 | 1 |
| CTTCA | 6317585 | 1.7467657 | 5.9016933 | 1 |
| TTTGA | 4553760 | 1.7416561 | 5.947172 | 2 |
| GGAAA | 4983520 | 1.325067 | 5.9301915 | 1 |
| GTTTG | 2802035 | 1.2730074 | 7.131133 | 1 |
| GTTTT | 2389560 | 1.0926023 | 5.744245 | 1 |

Produced by FastQC (version 0.10.1)
