# Supplementary material for: Meat Quality Characteristics of Mongolian Horses in Inner Mongolia: Regional Superiority and Transcriptomic Insights into Tenderness Differences Between Muscular Locations
Source: Animals (Basel). 2026 Jun 9;16(12):1788. doi: 10.3390/ani16121788 (PMC13295530; doi:10.3390/ani16121788)
Supplement: Supplementary file 1 [file animals-16-01788-s001.zip › TB2 R1.fq_fastqc.zip!/H6_TB_Clean_Data1.fq_fastqc/fastqc_report.html]

H6\_TB\_Clean\_Data1.fq.gz FastQC Report


FastQC Report

Thu 17 Dec 2020  
H6\_TB\_Clean\_Data1.fq.gz

## Summary

- Basic Statistics
- Per base sequence quality
- Per sequence quality scores
- Per base sequence content
- Per base GC content
- Per sequence GC content
- Per base N content
- Sequence Length Distribution
- Sequence Duplication Levels
- Overrepresented sequences
- Kmer Content

## Basic Statistics

| Measure | Value |
| --- | --- |
| Filename | H6\_TB\_Clean\_Data1.fq.gz |
| File type | Conventional base calls |
| Encoding | Sanger / Illumina 1.9 |
| Total Sequences | 25935234 |
| Filtered Sequences | 0 |
| Sequence length | 92-141 |
| %GC | 50 |

## Per base sequence quality

## Per sequence quality scores

## Per base sequence content

## Per base GC content

## Per sequence GC content

## Per base N content

## Sequence Length Distribution

## Sequence Duplication Levels

## Overrepresented sequences

| Sequence | Count | Percentage | Possible Source |
| --- | --- | --- | --- |
| GTCAGTATCAAGCGGCTGCTTCGAATCCGAAGTGGTGGCTGGATGTAAAG | 46855 | 0.1806615664234994 | No Hit |
| GTTTATTGATAGTTGTGTTGTTGGTGTAAATGAGTGAGGTAGTAGGCCTA | 45703 | 0.17621973258463755 | No Hit |
| GTTTGATATTGGGATAGAGCTGGTGGTTTTATGTTAATGATTGTGGTAAT | 35042 | 0.13511349078246218 | No Hit |
| GGCGATTGTTGATTAGTCGGTTGGGTGAGGGGAATAGGATGCTGGGAAAT | 33006 | 0.1272631663936404 | No Hit |
| GCTGGATGTAAAGTGGAATTTTAGTTGGCGTAGGAAACATACAATGAGGA | 32092 | 0.1237390030874601 | No Hit |
| GTTCGATTCCTTCCTTTCTTATTTTAGGTTTACGTAGGTGGGTTCTTCAA | 31149 | 0.1201030227836001 | No Hit |
| GTGGATTTTTGCTCAGGTTTGGTTGAGTGTGTACCCTGAGAATAGAGGGA | 27667 | 0.10667727154495694 | No Hit |
| GTTGTATATGCGTCTGGATAATCAGAATAGCGTCGTGGCATTCCTGAGAG | 27332 | 0.10538559243382958 | No Hit |
| GTCCTTTGCTGTTATGGATAGCTATTATTTGTTTTGATGTAAGTTGGACT | 26975 | 0.10400908663480729 | No Hit |

## Kmer Content

| Sequence | Count | Obs/Exp Overall | Obs/Exp Max | Max Obs/Exp Position |
| --- | --- | --- | --- | --- |
| CTCCT | 12484960 | 3.965752 | 8.910002 | 1 |
| CCTCC | 9395465 | 3.469823 | 4.3806677 | 3 |
| CTCCA | 9188885 | 3.4476914 | 9.862655 | 1 |
| TCCTC | 10693060 | 3.3965685 | 8.633306 | 2 |
| CTTCT | 11851650 | 3.237923 | 5.8884397 | 6 |
| CAGCT | 9423800 | 3.042746 | 4.656906 | 9 |
| TCCAG | 8880105 | 2.8671985 | 7.5554123 | 2 |
| TCTTC | 9939740 | 2.7155807 | 5.869306 | 5 |
| CTTCA | 7973035 | 2.5729928 | 6.0329375 | 6 |
| GCTCC | 7619460 | 2.4215157 | 8.02241 | 1 |
| TCCTT | 7914180 | 2.1621888 | 5.760517 | 2 |
| TCCAC | 5661810 | 2.1243246 | 6.2889752 | 2 |
| GCCAG | 6146950 | 1.9857478 | 6.617506 | 1 |
| GCTGG | 7787475 | 1.83277 | 8.057261 | 1 |
| GGCAG | 6389955 | 1.7763816 | 6.917709 | 1 |
| GCTCA | 4882655 | 1.576506 | 5.0249534 | 1 |
| GTTGA | 6478360 | 1.548204 | 7.951447 | 1 |
| GGGGA | 6261755 | 1.4979889 | 6.6239724 | 1 |
| GTCCA | 4437795 | 1.4328701 | 10.094916 | 1 |
| GGGAA | 5068450 | 1.4314961 | 5.496954 | 1 |
| TTTTT | 7001930 | 1.4151554 | 5.7180524 | 2 |
| GTCCT | 5172335 | 1.4138361 | 8.856375 | 1 |
| GGGGG | 6871810 | 1.3924553 | 6.6159697 | 1 |
| GTCAG | 4836105 | 1.3437221 | 9.901208 | 1 |
| GTGGG | 6572275 | 1.3310708 | 7.8614087 | 1 |
| GTGGA | 4931285 | 1.1790928 | 6.9922967 | 1 |
| GTTGG | 5816330 | 1.1773611 | 8.284393 | 1 |
| GTGCT | 4709800 | 1.1078706 | 6.365534 | 1 |
| GTCTG | 4699955 | 1.1055547 | 5.7676067 | 1 |
| GTCCC | 3439025 | 1.0929452 | 5.309553 | 1 |
| GTCAA | 3149360 | 1.0330907 | 5.2285514 | 1 |
| GTTTG | 5014695 | 1.0145663 | 6.158768 | 1 |
| GTTTA | 3022415 | 0.7219256 | 5.0493703 | 1 |

Produced by FastQC (version 0.10.1)
