# Supplementary material for: Meat Quality Characteristics of Mongolian Horses in Inner Mongolia: Regional Superiority and Transcriptomic Insights into Tenderness Differences Between Muscular Locations
Source: Animals (Basel). 2026 Jun 9;16(12):1788. doi: 10.3390/ani16121788 (PMC13295530; doi:10.3390/ani16121788)
Supplement: Supplementary file 1 [file animals-16-01788-s001.zip › TB2 R2.fq_fastqc.zip!/H6_TB_Clean_Data2.fq_fastqc/fastqc_report.html]

H6\_TB\_Clean\_Data2.fq.gz FastQC Report


FastQC Report

Thu 17 Dec 2020  
H6\_TB\_Clean\_Data2.fq.gz

## Summary

- Basic Statistics
- Per base sequence quality
- Per sequence quality scores
- Per base sequence content
- Per base GC content
- Per sequence GC content
- Per base N content
- Sequence Length Distribution
- Sequence Duplication Levels
- Overrepresented sequences
- Kmer Content

## Basic Statistics

| Measure | Value |
| --- | --- |
| Filename | H6\_TB\_Clean\_Data2.fq.gz |
| File type | Conventional base calls |
| Encoding | Sanger / Illumina 1.9 |
| Total Sequences | 25935234 |
| Filtered Sequences | 0 |
| Sequence length | 92-141 |
| %GC | 50 |

## Per base sequence quality

## Per sequence quality scores

## Per base sequence content

## Per base GC content

## Per sequence GC content

## Per base N content

## Sequence Length Distribution

## Sequence Duplication Levels

## Overrepresented sequences

| Sequence | Count | Percentage | Possible Source |
| --- | --- | --- | --- |
| CAGGAATCGTCCTAGCTAACTCATCCCTAGATATTGTTCTCCACGATACT | 29453 | 0.11356365629860907 | No Hit |
| GTAAACCCCAGCCCATGACCACTTACAGGAGCCCTATCAGCCCTCCTGAT | 27614 | 0.106472916342301 | No Hit |

## Kmer Content

| Sequence | Count | Obs/Exp Overall | Obs/Exp Max | Max Obs/Exp Position |
| --- | --- | --- | --- | --- |
| AGGAG | 12556565 | 3.8240633 | 4.825323 | 1 |
| GGAGG | 9435995 | 3.343323 | 5.800815 | 1 |
| TGGAG | 8981360 | 3.2793722 | 4.9140615 | 7 |
| GAGGA | 10665215 | 3.2480583 | 5.7922564 | 1 |
| AGAAG | 12003990 | 3.14227 | 5.002394 | 3 |
| AGCTG | 9377960 | 3.0030913 | 3.9453542 | 6 |
| GAAGA | 10131215 | 2.6520355 | 6.2448726 | 2 |
| GGAGA | 8391825 | 2.5557046 | 7.362508 | 1 |
| GCTGA | 6986960 | 2.2374246 | 5.0515738 | 1 |
| GAGAA | 8190345 | 2.1439764 | 5.222219 | 2 |
| GTGGA | 5575335 | 2.0357273 | 6.9199204 | 1 |
| GGAAG | 6218245 | 1.8937473 | 5.922264 | 1 |
| CTTCA | 6285665 | 1.819202 | 5.5947213 | 1 |
| TTTGA | 4505385 | 1.747014 | 6.3018246 | 2 |
| GGAAA | 5166630 | 1.3524623 | 6.1549516 | 1 |
| GTTTG | 2867835 | 1.293764 | 7.925061 | 1 |
| GTGAA | 3755720 | 1.1787057 | 5.2280016 | 1 |
| GTTTT | 2413330 | 1.1219554 | 6.343916 | 1 |
| GTTCT | 2578535 | 1.0202004 | 5.2015705 | 1 |
| GTTGA | 2331580 | 0.87731767 | 5.3238225 | 1 |

Produced by FastQC (version 0.10.1)
