# Supplementary material for: Meat Quality Characteristics of Mongolian Horses in Inner Mongolia: Regional Superiority and Transcriptomic Insights into Tenderness Differences Between Muscular Locations
Source: Animals (Basel). 2026 Jun 9;16(12):1788. doi: 10.3390/ani16121788 (PMC13295530; doi:10.3390/ani16121788)
Supplement: Supplementary file 1 [file animals-16-01788-s001.zip › TB3 R1.fq_fastqc.zip!/H10_TB_Clean_Data1.fq_fastqc/fastqc_report.html]

H10\_TB\_Clean\_Data1.fq.gz FastQC Report


FastQC Report

Thu 17 Dec 2020  
H10\_TB\_Clean\_Data1.fq.gz

## Summary

- Basic Statistics
- Per base sequence quality
- Per sequence quality scores
- Per base sequence content
- Per base GC content
- Per sequence GC content
- Per base N content
- Sequence Length Distribution
- Sequence Duplication Levels
- Overrepresented sequences
- Kmer Content

## Basic Statistics

| Measure | Value |
| --- | --- |
| Filename | H10\_TB\_Clean\_Data1.fq.gz |
| File type | Conventional base calls |
| Encoding | Sanger / Illumina 1.9 |
| Total Sequences | 24758664 |
| Filtered Sequences | 0 |
| Sequence length | 92-141 |
| %GC | 50 |

## Per base sequence quality

## Per sequence quality scores

## Per base sequence content

## Per base GC content

## Per sequence GC content

## Per base N content

## Sequence Length Distribution

## Sequence Duplication Levels

## Overrepresented sequences

| Sequence | Count | Percentage | Possible Source |
| --- | --- | --- | --- |
| GTCAGTATCAAGCGGCTGCTTCGAATCCGAAGTGGTGGCTGGATGTAAAG | 39051 | 0.15772660431112115 | No Hit |
| GGCGATTGTTGATTAGTCGGTTGGGTGAGGGGAATAGGATGCTGGGAAAT | 36568 | 0.14769779177099376 | No Hit |
| GTTTATTGATAGTTGTGTTGTTGGTGTAAATGAGTGAGGTAGTAGGCCTA | 34973 | 0.14125560248323576 | No Hit |
| GTTTGATATTGGGATAGGGCTGGTGGTTTTATGTTAATGATTGTGGTAAT | 30859 | 0.12463919700998406 | No Hit |
| GTTCGATTCCTTCCTTTCTTATTTTAGGTTTACGTAGGTGGGTTCTTCAA | 28135 | 0.11363698784393211 | No Hit |
| GTCCTTTGCTGTTATGGATAGCTATTATTTGTTTTGATGTAAGTTGGACT | 26001 | 0.1050177828658283 | No Hit |
| GTGAGATTATACCGAAGCCTGGTAGGATAAGAATATAGACTTCGGGGTGT | 25818 | 0.10427864766854948 | No Hit |
| GCTGGATGTAAAGTGGAATTTTAGTTGGCGTAGGAAACATACAATGAGGA | 25474 | 0.10288923505727125 | No Hit |

## Kmer Content

| Sequence | Count | Obs/Exp Overall | Obs/Exp Max | Max Obs/Exp Position |
| --- | --- | --- | --- | --- |
| CTCCT | 11831330 | 3.9355965 | 9.962541 | 1 |
| CCTCC | 9007220 | 3.470166 | 4.286315 | 3 |
| TCCTC | 10302060 | 3.426897 | 8.580522 | 2 |
| CTCCA | 8669080 | 3.3900406 | 10.700474 | 1 |
| CTTCT | 11435225 | 3.284272 | 5.6862354 | 6 |
| TCCAG | 8428340 | 2.8332343 | 7.247493 | 2 |
| TCTTC | 9595185 | 2.7558002 | 5.7047234 | 7 |
| CTTCA | 7715610 | 2.6050713 | 5.985319 | 6 |
| GCTCC | 7192455 | 2.3820193 | 7.8973265 | 1 |
| TTCTT | 9450880 | 2.343603 | 5.104602 | 6 |
| TCCTT | 7638060 | 2.193701 | 6.108187 | 2 |
| TCCAC | 5425840 | 2.1217728 | 6.102532 | 2 |
| CTCAG | 6103955 | 2.0518792 | 5.190434 | 1 |
| GCCAG | 6002810 | 2.009029 | 6.303898 | 1 |
| GCTTC | 6540955 | 1.8703663 | 5.0369935 | 1 |
| GCTGG | 7530825 | 1.8430104 | 7.9085164 | 1 |
| GGCAG | 6302805 | 1.813318 | 6.8012176 | 1 |
| GGGGA | 6162690 | 1.5241195 | 6.2635636 | 1 |
| GTTGA | 6032460 | 1.5050845 | 7.280165 | 1 |
| TTTTT | 6976000 | 1.493605 | 5.923581 | 2 |
| GGGGG | 7098940 | 1.4868884 | 6.530338 | 1 |
| GGGAA | 5028260 | 1.4683522 | 5.2177906 | 1 |
| GTCCA | 4092645 | 1.3757657 | 9.149245 | 1 |
| GTCCT | 4789925 | 1.3696644 | 8.488494 | 1 |
| GTGGG | 6433355 | 1.3534158 | 7.786652 | 1 |
| GTCAG | 4512880 | 1.3040756 | 8.640359 | 1 |
| GTTGG | 5581740 | 1.1794302 | 7.7656946 | 1 |
| GTGGA | 4711880 | 1.170447 | 6.7615337 | 1 |
| GTCTG | 4542885 | 1.1166724 | 5.1078887 | 1 |
| GTGCT | 4456225 | 1.0953709 | 5.8970656 | 1 |
| GTCCC | 3297140 | 1.0919567 | 5.29664 | 1 |
| GTTTG | 4686200 | 0.99456304 | 5.6437545 | 1 |

Produced by FastQC (version 0.10.1)
