# Supplementary material for: Meat Quality Characteristics of Mongolian Horses in Inner Mongolia: Regional Superiority and Transcriptomic Insights into Tenderness Differences Between Muscular Locations
Source: Animals (Basel). 2026 Jun 9;16(12):1788. doi: 10.3390/ani16121788 (PMC13295530; doi:10.3390/ani16121788)
Supplement: Supplementary file 1 [file animals-16-01788-s001.zip › TB3 R2.fq_fastqc.zip!/H10_TB_Clean_Data2.fq_fastqc/fastqc_report.html]

H10\_TB\_Clean\_Data2.fq.gz FastQC Report


FastQC Report

Thu 17 Dec 2020  
H10\_TB\_Clean\_Data2.fq.gz

## Summary

- Basic Statistics
- Per base sequence quality
- Per sequence quality scores
- Per base sequence content
- Per base GC content
- Per sequence GC content
- Per base N content
- Sequence Length Distribution
- Sequence Duplication Levels
- Overrepresented sequences
- Kmer Content

## Basic Statistics

| Measure | Value |
| --- | --- |
| Filename | H10\_TB\_Clean\_Data2.fq.gz |
| File type | Conventional base calls |
| Encoding | Sanger / Illumina 1.9 |
| Total Sequences | 24758664 |
| Filtered Sequences | 0 |
| Sequence length | 92-141 |
| %GC | 50 |

## Per base sequence quality

## Per sequence quality scores

## Per base sequence content

## Per base GC content

## Per sequence GC content

## Per base N content

## Sequence Length Distribution

## Sequence Duplication Levels

## Overrepresented sequences

| Sequence | Count | Percentage | Possible Source |
| --- | --- | --- | --- |
| CAGGAATCGTCCTAGCTAACTCATCCCTAGATATTGTTCTCCACGATACT | 25712 | 0.10385051471274863 | No Hit |

## Kmer Content

| Sequence | Count | Obs/Exp Overall | Obs/Exp Max | Max Obs/Exp Position |
| --- | --- | --- | --- | --- |
| AGGAG | 11873115 | 3.7885973 | 4.3551884 | 2 |
| GGAGG | 9035865 | 3.3350806 | 5.620044 | 1 |
| GAGGA | 10298215 | 3.2860618 | 5.8658314 | 1 |
| TGGAG | 8502880 | 3.2277842 | 4.8907676 | 7 |
| AGAAG | 11606530 | 3.201794 | 5.096497 | 3 |
| GAAGA | 9788820 | 2.7003577 | 6.102625 | 2 |
| GGAGA | 8064820 | 2.573407 | 6.988543 | 1 |
| GAGAA | 7852610 | 2.166232 | 5.1170297 | 2 |
| TTTTT | 4347340 | 2.1359556 | 5.3792086 | 2 |
| GTGGA | 5397935 | 2.0491138 | 6.750802 | 1 |
| GGAAG | 6072310 | 1.937616 | 5.7808843 | 1 |
| CTTCA | 5899065 | 1.7726392 | 5.512103 | 1 |
| TTTGA | 4337790 | 1.7418495 | 5.7055297 | 2 |
| GGAAA | 5038205 | 1.3898463 | 6.102995 | 1 |
| GTTTG | 2774345 | 1.2886214 | 7.230644 | 1 |
| GTTTT | 2450905 | 1.1708282 | 6.394612 | 1 |

Produced by FastQC (version 0.10.1)
