# Supplementary material for: Meat Quality Characteristics of Mongolian Horses in Inner Mongolia: Regional Superiority and Transcriptomic Insights into Tenderness Differences Between Muscular Locations
Source: Animals (Basel). 2026 Jun 9;16(12):1788. doi: 10.3390/ani16121788 (PMC13295530; doi:10.3390/ani16121788)
Supplement: Supplementary file 1 [file animals-16-01788-s001.zip › TB4 R1.fq_fastqc.zip!/H11_TB_Clean_Data1.fq_fastqc/fastqc_report.html]

H11\_TB\_Clean\_Data1.fq.gz FastQC Report


FastQC Report

Thu 17 Dec 2020  
H11\_TB\_Clean\_Data1.fq.gz

## Summary

- Basic Statistics
- Per base sequence quality
- Per sequence quality scores
- Per base sequence content
- Per base GC content
- Per sequence GC content
- Per base N content
- Sequence Length Distribution
- Sequence Duplication Levels
- Overrepresented sequences
- Kmer Content

## Basic Statistics

| Measure | Value |
| --- | --- |
| Filename | H11\_TB\_Clean\_Data1.fq.gz |
| File type | Conventional base calls |
| Encoding | Sanger / Illumina 1.9 |
| Total Sequences | 25423202 |
| Filtered Sequences | 0 |
| Sequence length | 92-141 |
| %GC | 50 |

## Per base sequence quality

## Per sequence quality scores

## Per base sequence content

## Per base GC content

## Per sequence GC content

## Per base N content

## Sequence Length Distribution

## Sequence Duplication Levels

## Overrepresented sequences

| Sequence | Count | Percentage | Possible Source |
| --- | --- | --- | --- |
| GTCAGTATCAAGCGGCTGCTTCGAATCCGAAGTGGTGGCTGGATGTAAAG | 37678 | 0.14820320430133072 | No Hit |
| GTTTCATGTTGTATATGCGTCTGGATAATCAGAATAGCGTCGTGGCATTC | 35201 | 0.1384601357452928 | No Hit |
| GTTTATTGATAGTTGTGTTGTTGGTGTGAATGAGTGAGGTAGTAGGCCTA | 29149 | 0.1146551091400682 | No Hit |
| GGCGATTGTTAATTAGTCGGTTGGGTGAGGGGAATAGGATGCTGGGAAAT | 25661 | 0.10093535818186868 | No Hit |

## Kmer Content

| Sequence | Count | Obs/Exp Overall | Obs/Exp Max | Max Obs/Exp Position |
| --- | --- | --- | --- | --- |
| CTCCT | 12665290 | 3.9014926 | 11.081338 | 1 |
| CTCCA | 9486515 | 3.4590595 | 11.754511 | 1 |
| TCCTC | 10897850 | 3.3570397 | 8.943562 | 2 |
| CCTCC | 9574415 | 3.3380656 | 4.4631605 | 3 |
| CTTCT | 11998560 | 3.2657118 | 5.6286182 | 6 |
| CAGCT | 9561110 | 3.082071 | 4.5353703 | 9 |
| TCCAG | 9462370 | 3.0502417 | 8.181066 | 2 |
| TCTTC | 10066920 | 2.739967 | 5.8360915 | 5 |
| CTTCA | 7933800 | 2.5560281 | 5.7351284 | 6 |
| TTCTT | 9742030 | 2.3427784 | 5.0233426 | 6 |
| GCTCC | 7466740 | 2.3014243 | 7.443499 | 1 |
| TCCTT | 8268875 | 2.2505836 | 5.840988 | 2 |
| TCCAC | 5862560 | 2.13766 | 6.000923 | 2 |
| CTCAG | 6111320 | 1.9700141 | 5.6424174 | 1 |
| GCCAG | 6074815 | 1.959374 | 6.006727 | 1 |
| GCTGG | 7697415 | 1.8542851 | 7.336073 | 1 |
| GGCAG | 6473280 | 1.84583 | 6.8909364 | 1 |
| GGGGA | 6109530 | 1.5401326 | 5.9258947 | 1 |
| GTCCA | 4707555 | 1.5175036 | 8.995133 | 1 |
| TTTTT | 7038330 | 1.4954932 | 5.8558936 | 2 |
| GTTGA | 5727490 | 1.4421642 | 6.2747684 | 1 |
| GGGAA | 4736930 | 1.4126451 | 5.0580072 | 1 |
| GGGGG | 6601610 | 1.406742 | 5.8545947 | 1 |
| GTCCT | 5001245 | 1.3619992 | 7.555539 | 1 |
| GTGGG | 6382565 | 1.359283 | 7.076304 | 1 |
| GTCAG | 4759905 | 1.3564869 | 9.076606 | 1 |
| GTTGG | 5618480 | 1.1958687 | 7.4774256 | 1 |
| GTGGA | 4597005 | 1.158178 | 5.8105116 | 1 |
| GTGCT | 4578110 | 1.1022189 | 5.720715 | 1 |
| GTTTG | 4539780 | 0.9657163 | 5.026921 | 1 |

Produced by FastQC (version 0.10.1)
