# Supplementary material for: Meat Quality Characteristics of Mongolian Horses in Inner Mongolia: Regional Superiority and Transcriptomic Insights into Tenderness Differences Between Muscular Locations
Source: Animals (Basel). 2026 Jun 9;16(12):1788. doi: 10.3390/ani16121788 (PMC13295530; doi:10.3390/ani16121788)
Supplement: Supplementary file 1 [file animals-16-01788-s001.zip › TB4 R2.fq_fastqc.zip!/H11_TB_Clean_Data2.fq_fastqc/fastqc_report.html]

H11\_TB\_Clean\_Data2.fq.gz FastQC Report


FastQC Report

Thu 17 Dec 2020  
H11\_TB\_Clean\_Data2.fq.gz

## Summary

- Basic Statistics
- Per base sequence quality
- Per sequence quality scores
- Per base sequence content
- Per base GC content
- Per sequence GC content
- Per base N content
- Sequence Length Distribution
- Sequence Duplication Levels
- Overrepresented sequences
- Kmer Content

## Basic Statistics

| Measure | Value |
| --- | --- |
| Filename | H11\_TB\_Clean\_Data2.fq.gz |
| File type | Conventional base calls |
| Encoding | Sanger / Illumina 1.9 |
| Total Sequences | 25423202 |
| Filtered Sequences | 0 |
| Sequence length | 92-141 |
| %GC | 50 |

## Per base sequence quality

## Per sequence quality scores

## Per base sequence content

## Per base GC content

## Per sequence GC content

## Per base N content

## Sequence Length Distribution

## Sequence Duplication Levels

## Overrepresented sequences

No overrepresented sequences

## Kmer Content

| Sequence | Count | Obs/Exp Overall | Obs/Exp Max | Max Obs/Exp Position |
| --- | --- | --- | --- | --- |
| AGGAG | 12633675 | 3.757337 | 4.2963147 | 2 |
| TGGAG | 9248820 | 3.3102615 | 4.953815 | 7 |
| GGAGG | 9609390 | 3.2459345 | 5.170247 | 1 |
| GAGGA | 10879690 | 3.2356906 | 5.4576416 | 1 |
| AGAAG | 12117720 | 3.1730576 | 5.1866302 | 3 |
| AGCTG | 9502210 | 3.0476677 | 4.0256686 | 6 |
| GAAGA | 10336055 | 2.7065241 | 6.2485595 | 2 |
| GGAGA | 8708915 | 2.5900881 | 6.4590387 | 1 |
| TTTTT | 4366685 | 2.2374299 | 5.665377 | 2 |
| GAGAA | 8251270 | 2.1606169 | 5.018424 | 2 |
| GTGGA | 5764905 | 2.0633273 | 6.365477 | 1 |
| GGAAG | 6406605 | 1.9053662 | 5.5656495 | 1 |
| CTTCA | 6012330 | 1.8309855 | 5.7453833 | 1 |
| TTTGA | 4428480 | 1.7794864 | 5.880286 | 2 |
| GGAAA | 5072445 | 1.3282335 | 5.4993114 | 1 |
| GTTTG | 2886275 | 1.3172591 | 6.8371453 | 1 |
| GTTTT | 2571490 | 1.2435131 | 6.49109 | 1 |

Produced by FastQC (version 0.10.1)
