# Supplementary material for: Meat Quality Characteristics of Mongolian Horses in Inner Mongolia: Regional Superiority and Transcriptomic Insights into Tenderness Differences Between Muscular Locations
Source: Animals (Basel). 2026 Jun 9;16(12):1788. doi: 10.3390/ani16121788 (PMC13295530; doi:10.3390/ani16121788)
Supplement: Supplementary file 1 [file animals-16-01788-s001.zip › TB5 R1.fq_fastqc.zip!/H13_TB_Clean_Data1.fq_fastqc/fastqc_report.html]

H13\_TB\_Clean\_Data1.fq.gz FastQC Report


FastQC Report

Thu 17 Dec 2020  
H13\_TB\_Clean\_Data1.fq.gz

## Summary

- Basic Statistics
- Per base sequence quality
- Per sequence quality scores
- Per base sequence content
- Per base GC content
- Per sequence GC content
- Per base N content
- Sequence Length Distribution
- Sequence Duplication Levels
- Overrepresented sequences
- Kmer Content

## Basic Statistics

| Measure | Value |
| --- | --- |
| Filename | H13\_TB\_Clean\_Data1.fq.gz |
| File type | Conventional base calls |
| Encoding | Sanger / Illumina 1.9 |
| Total Sequences | 27175038 |
| Filtered Sequences | 0 |
| Sequence length | 92-141 |
| %GC | 50 |

## Per base sequence quality

## Per sequence quality scores

## Per base sequence content

## Per base GC content

## Per sequence GC content

## Per base N content

## Sequence Length Distribution

## Sequence Duplication Levels

## Overrepresented sequences

| Sequence | Count | Percentage | Possible Source |
| --- | --- | --- | --- |
| GTTTATTGATAGTTGTGTTGTTGGTGTAAATGAGTGAGGTAGTAGGCCTA | 39074 | 0.14378636747444476 | No Hit |
| GTCAGTATCAAGCGGCTGCTTCGAATCCGAAGTGGTGGCTGGATGTAAAG | 37113 | 0.1365701862128031 | No Hit |
| GTTTGATATTGGGATAGGGCTGGTGGTTTTATGTTAATGATTGTGGTAAT | 30803 | 0.113350347476975 | No Hit |
| GGCGATTGTTGATTAGTCGGTTGGGTGAGGGGAATAGGATGCTGGGAAAT | 30727 | 0.11307067905480023 | No Hit |

## Kmer Content

| Sequence | Count | Obs/Exp Overall | Obs/Exp Max | Max Obs/Exp Position |
| --- | --- | --- | --- | --- |
| CTCCT | 12920220 | 3.7385237 | 8.555342 | 1 |
| CTCCA | 9673060 | 3.2969038 | 9.807701 | 1 |
| TCCTC | 11342380 | 3.2819686 | 8.150978 | 2 |
| CCTCC | 9963380 | 3.245584 | 3.9496744 | 3 |
| CTTCT | 12527845 | 3.219962 | 5.7666016 | 6 |
| TCCAG | 9374870 | 2.8074098 | 7.206547 | 2 |
| TCTTC | 10663830 | 2.7408645 | 5.810594 | 5 |
| CTTCA | 8570350 | 2.5946875 | 5.939661 | 6 |
| TTCTT | 10409180 | 2.3764846 | 5.172246 | 6 |
| GCTCC | 7994480 | 2.2880976 | 7.481647 | 1 |
| TCCTT | 8533290 | 2.1932638 | 5.8004155 | 2 |
| TCCAC | 6259730 | 2.1335263 | 6.475174 | 2 |
| GCCAG | 6883320 | 2.0388842 | 6.8266163 | 1 |
| GGCAG | 7259475 | 1.8892869 | 7.29342 | 1 |
| GCTGG | 8470670 | 1.8715365 | 8.287608 | 1 |
| GCTTC | 7252975 | 1.8439306 | 5.0302277 | 1 |
| GGGGA | 6606895 | 1.5107349 | 6.5482283 | 1 |
| GTTGA | 6436240 | 1.5042326 | 7.474298 | 1 |
| TTTTT | 7334930 | 1.4875053 | 6.447759 | 2 |
| GGGGG | 7503745 | 1.4408234 | 6.4543056 | 1 |
| GGGAA | 5290140 | 1.4405093 | 5.5244727 | 1 |
| GTCCA | 4751330 | 1.4228389 | 9.947018 | 1 |
| GTCCT | 5490395 | 1.3958282 | 8.610668 | 1 |
| GTGGG | 6854355 | 1.3305931 | 7.671593 | 1 |
| GTCAG | 5016345 | 1.3198545 | 9.036106 | 1 |
| GTTGG | 5915345 | 1.1609265 | 7.923844 | 1 |
| GTGGA | 4983270 | 1.1519967 | 6.784656 | 1 |
| GTCAT | 4306795 | 1.1456153 | 5.019593 | 1 |
| GTCTG | 4970610 | 1.1102895 | 5.538104 | 1 |
| GTGCT | 4935515 | 1.1024503 | 6.1576133 | 1 |
| GTCCC | 3734310 | 1.0687957 | 5.0804887 | 1 |
| GTTTG | 4979590 | 0.9880165 | 5.8635516 | 1 |
| GTCAC | 3198090 | 0.9577038 | 5.0865726 | 1 |

Produced by FastQC (version 0.10.1)
