# Supplementary material for: Meat Quality Characteristics of Mongolian Horses in Inner Mongolia: Regional Superiority and Transcriptomic Insights into Tenderness Differences Between Muscular Locations
Source: Animals (Basel). 2026 Jun 9;16(12):1788. doi: 10.3390/ani16121788 (PMC13295530; doi:10.3390/ani16121788)
Supplement: Supplementary file 1 [file animals-16-01788-s001.zip › TB5 R2.fq_fastqc.zip!/H13_TB_Clean_Data2.fq_fastqc/fastqc_report.html]

H13\_TB\_Clean\_Data2.fq.gz FastQC Report


FastQC Report

Thu 17 Dec 2020  
H13\_TB\_Clean\_Data2.fq.gz

## Summary

- Basic Statistics
- Per base sequence quality
- Per sequence quality scores
- Per base sequence content
- Per base GC content
- Per sequence GC content
- Per base N content
- Sequence Length Distribution
- Sequence Duplication Levels
- Overrepresented sequences
- Kmer Content

## Basic Statistics

| Measure | Value |
| --- | --- |
| Filename | H13\_TB\_Clean\_Data2.fq.gz |
| File type | Conventional base calls |
| Encoding | Sanger / Illumina 1.9 |
| Total Sequences | 27175038 |
| Filtered Sequences | 0 |
| Sequence length | 92-141 |
| %GC | 50 |

## Per base sequence quality

## Per sequence quality scores

## Per base sequence content

## Per base GC content

## Per sequence GC content

## Per base N content

## Sequence Length Distribution

## Sequence Duplication Levels

## Overrepresented sequences

No overrepresented sequences

## Kmer Content

| Sequence | Count | Obs/Exp Overall | Obs/Exp Max | Max Obs/Exp Position |
| --- | --- | --- | --- | --- |
| AGGAG | 13008090 | 3.6039748 | 4.405623 | 1 |
| GAGGA | 11374925 | 3.151496 | 5.737538 | 1 |
| TGGAG | 9502320 | 3.1374745 | 4.8270574 | 7 |
| AGAAG | 12663895 | 3.1308832 | 4.9615316 | 3 |
| GGAGG | 10011940 | 3.1085303 | 5.469416 | 1 |
| GAAGA | 10841950 | 2.6804454 | 6.2532754 | 2 |
| GGAGA | 9019200 | 2.4988272 | 7.296336 | 1 |
| TTTTT | 4616130 | 2.1844993 | 5.4959683 | 2 |
| GAGAA | 8615885 | 2.1300974 | 5.1541977 | 2 |
| GTGGA | 6221580 | 2.0542402 | 6.9185605 | 1 |
| GGAAG | 6918110 | 1.9167067 | 6.0566616 | 1 |
| CTTCA | 6544515 | 1.84977 | 5.555775 | 1 |
| TTTGA | 4678620 | 1.7470111 | 5.933171 | 2 |
| GGAAA | 5591690 | 1.3824283 | 6.303059 | 1 |
| GTTTG | 3087515 | 1.2919796 | 7.5750484 | 1 |
| GTGAA | 4216885 | 1.2424349 | 5.246981 | 1 |
| GTTTT | 2717825 | 1.2094327 | 6.960292 | 1 |
| GTTCT | 2760725 | 1.0364723 | 5.2132206 | 1 |
| GTTGA | 2484345 | 0.87232065 | 5.1111393 | 1 |

Produced by FastQC (version 0.10.1)
