# Supplementary material for: Meat Quality Characteristics of Mongolian Horses in Inner Mongolia: Regional Superiority and Transcriptomic Insights into Tenderness Differences Between Muscular Locations
Source: Animals (Basel). 2026 Jun 9;16(12):1788. doi: 10.3390/ani16121788 (PMC13295530; doi:10.3390/ani16121788)
Supplement: Supplementary file 1 [file animals-16-01788-s001.zip › TB6 R1.fq_fastqc.zip!/H14_TB_Clean_Data1.fq_fastqc/fastqc_report.html]

H14\_TB\_Clean\_Data1.fq.gz FastQC Report


FastQC Report

Thu 17 Dec 2020  
H14\_TB\_Clean\_Data1.fq.gz

## Summary

- Basic Statistics
- Per base sequence quality
- Per sequence quality scores
- Per base sequence content
- Per base GC content
- Per sequence GC content
- Per base N content
- Sequence Length Distribution
- Sequence Duplication Levels
- Overrepresented sequences
- Kmer Content

## Basic Statistics

| Measure | Value |
| --- | --- |
| Filename | H14\_TB\_Clean\_Data1.fq.gz |
| File type | Conventional base calls |
| Encoding | Sanger / Illumina 1.9 |
| Total Sequences | 25272697 |
| Filtered Sequences | 0 |
| Sequence length | 92-141 |
| %GC | 51 |

## Per base sequence quality

## Per sequence quality scores

## Per base sequence content

## Per base GC content

## Per sequence GC content

## Per base N content

## Sequence Length Distribution

## Sequence Duplication Levels

## Overrepresented sequences

| Sequence | Count | Percentage | Possible Source |
| --- | --- | --- | --- |
| GTTTGATATTGGGATAGAGCCGGTGGTTTTATGTTAATGATTGTGGTAAT | 27867 | 0.11026523999397453 | No Hit |

## Kmer Content

| Sequence | Count | Obs/Exp Overall | Obs/Exp Max | Max Obs/Exp Position |
| --- | --- | --- | --- | --- |
| CTCCT | 12143510 | 3.4521265 | 4.7848754 | 1 |
| CTTCT | 11831235 | 3.1511014 | 5.652004 | 6 |
| CTCCA | 9514025 | 3.13887 | 5.3608913 | 6 |
| TCCTC | 10865470 | 3.0888085 | 4.9031606 | 2 |
| TCCAG | 9069375 | 2.7872093 | 5.3600903 | 7 |
| TCTTC | 10294425 | 2.7417912 | 5.325291 | 5 |
| CTTCA | 8364440 | 2.5854475 | 5.603735 | 6 |
| GCTCC | 7729160 | 2.1845808 | 6.4245696 | 1 |
| TCCAC | 6418040 | 2.1174417 | 5.335305 | 2 |
| GCCAG | 6807190 | 2.0799522 | 9.249881 | 1 |
| GGCAG | 7056935 | 2.00856 | 5.641939 | 1 |
| GCTGG | 7978610 | 1.956723 | 10.250066 | 1 |
| GCTTC | 7013270 | 1.8571465 | 6.3862867 | 1 |
| TTTTT | 7733435 | 1.8079412 | 8.121851 | 2 |
| GCCCA | 5133175 | 1.6837921 | 5.977961 | 1 |
| GGAGA | 5327850 | 1.6488345 | 6.1971893 | 1 |
| GGGAG | 6007790 | 1.5928193 | 5.4909406 | 1 |
| GGGGG | 6986520 | 1.586863 | 7.413068 | 1 |
| GGGGA | 5830135 | 1.5457184 | 8.192517 | 1 |
| GCCAC | 4588120 | 1.5050024 | 5.745249 | 1 |
| GTCCA | 4802205 | 1.4758182 | 9.599583 | 1 |
| GCTGA | 5087670 | 1.4564455 | 5.8607 | 1 |
| GCTCA | 4616205 | 1.4186565 | 6.1895022 | 1 |
| GGGAA | 4446770 | 1.3761624 | 5.257405 | 1 |
| GTCAG | 4784920 | 1.3697773 | 6.489087 | 1 |
| GTCCT | 5126220 | 1.3574469 | 5.00249 | 1 |
| GCCTG | 5138520 | 1.3528731 | 5.2146354 | 1 |
| GTGGG | 5846850 | 1.3356946 | 5.491422 | 1 |
| GTTGA | 4964745 | 1.3315641 | 6.523794 | 1 |
| GTGGA | 4333880 | 1.1556741 | 5.456908 | 1 |
| GTTGG | 4865775 | 1.1180053 | 7.2490473 | 1 |
| GTTTT | 4705055 | 1.0936286 | 6.2043023 | 1 |
| GTTCA | 3744255 | 1.0780711 | 5.694823 | 1 |
| GTTTG | 4239635 | 0.97977644 | 7.8014083 | 1 |
| GTTTA | 2620940 | 0.70701534 | 5.8981056 | 1 |

Produced by FastQC (version 0.10.1)
