# Supplementary material for: Meat Quality Characteristics of Mongolian Horses in Inner Mongolia: Regional Superiority and Transcriptomic Insights into Tenderness Differences Between Muscular Locations
Source: Animals (Basel). 2026 Jun 9;16(12):1788. doi: 10.3390/ani16121788 (PMC13295530; doi:10.3390/ani16121788)
Supplement: Supplementary file 1 [file animals-16-01788-s001.zip › TB6 R2.fq_fastqc.zip!/H14_TB_Clean_Data2.fq_fastqc/fastqc_report.html]

H14\_TB\_Clean\_Data2.fq.gz FastQC Report


FastQC Report

Thu 17 Dec 2020  
H14\_TB\_Clean\_Data2.fq.gz

## Summary

- Basic Statistics
- Per base sequence quality
- Per sequence quality scores
- Per base sequence content
- Per base GC content
- Per sequence GC content
- Per base N content
- Sequence Length Distribution
- Sequence Duplication Levels
- Overrepresented sequences
- Kmer Content

## Basic Statistics

| Measure | Value |
| --- | --- |
| Filename | H14\_TB\_Clean\_Data2.fq.gz |
| File type | Conventional base calls |
| Encoding | Sanger / Illumina 1.9 |
| Total Sequences | 25272697 |
| Filtered Sequences | 0 |
| Sequence length | 92-141 |
| %GC | 51 |

## Per base sequence quality

## Per sequence quality scores

## Per base sequence content

## Per base GC content

## Per sequence GC content

## Per base N content

## Sequence Length Distribution

## Sequence Duplication Levels

## Overrepresented sequences

No overrepresented sequences

## Kmer Content

| Sequence | Count | Obs/Exp Overall | Obs/Exp Max | Max Obs/Exp Position |
| --- | --- | --- | --- | --- |
| AGGAG | 12323340 | 3.3262398 | 3.8759391 | 2 |
| AGAAG | 11838310 | 3.054755 | 4.983493 | 3 |
| GAGGA | 11026220 | 2.976129 | 5.8761067 | 1 |
| GGAGG | 9655515 | 2.7260828 | 5.2994385 | 1 |
| GCTGG | 8624120 | 2.7221682 | 9.51839 | 1 |
| CTGGA | 8963940 | 2.704959 | 5.5523367 | 2 |
| GGAGA | 9012775 | 2.4326725 | 9.012287 | 1 |
| GCTGC | 7454765 | 2.2523313 | 5.3493495 | 1 |
| GAGAA | 8323735 | 2.1478548 | 5.2598124 | 2 |
| GCTGA | 7094625 | 2.1408741 | 10.2872505 | 1 |
| GTGGA | 6435110 | 2.0287085 | 7.9289665 | 1 |
| GGAAG | 6852225 | 1.8495102 | 5.876835 | 1 |
| CTGAA | 6373030 | 1.8385239 | 5.5339336 | 2 |
| TTTGA | 4603660 | 1.8095355 | 5.257656 | 2 |
| GCAGA | 6841100 | 1.7674582 | 6.0730085 | 1 |
| GCCAG | 5659475 | 1.4639826 | 6.6148314 | 1 |
| GTTTT | 3145485 | 1.4440758 | 5.704276 | 1 |
| GGGAG | 5083395 | 1.4352165 | 5.434809 | 1 |
| GCCAA | 5760905 | 1.4246627 | 6.411719 | 1 |
| GTTTG | 3167895 | 1.302486 | 8.539166 | 1 |
| GGGGA | 4549560 | 1.2844967 | 5.545238 | 1 |
| GTTCA | 3024910 | 1.0192349 | 7.351123 | 1 |
| GTTGA | 2338610 | 0.8232307 | 5.9595656 | 1 |
| GTTTA | 1961170 | 0.77086633 | 5.3029165 | 1 |

Produced by FastQC (version 0.10.1)
