# Supplementary material for: Meat Quality Characteristics of Mongolian Horses in Inner Mongolia: Regional Superiority and Transcriptomic Insights into Tenderness Differences Between Muscular Locations
Source: Animals (Basel). 2026 Jun 9;16(12):1788. doi: 10.3390/ani16121788 (PMC13295530; doi:10.3390/ani16121788)
Supplement: Supplementary file 1 [file animals-16-01788-s001.zip › TB7 R1.fq_fastqc.zip!/H15_TB_Clean_Data1.fq_fastqc/fastqc_report.html]

H15\_TB\_Clean\_Data1.fq.gz FastQC Report


FastQC Report

Thu 17 Dec 2020  
H15\_TB\_Clean\_Data1.fq.gz

## Summary

- Basic Statistics
- Per base sequence quality
- Per sequence quality scores
- Per base sequence content
- Per base GC content
- Per sequence GC content
- Per base N content
- Sequence Length Distribution
- Sequence Duplication Levels
- Overrepresented sequences
- Kmer Content

## Basic Statistics

| Measure | Value |
| --- | --- |
| Filename | H15\_TB\_Clean\_Data1.fq.gz |
| File type | Conventional base calls |
| Encoding | Sanger / Illumina 1.9 |
| Total Sequences | 25941255 |
| Filtered Sequences | 0 |
| Sequence length | 92-141 |
| %GC | 51 |

## Per base sequence quality

## Per sequence quality scores

## Per base sequence content

## Per base GC content

## Per sequence GC content

## Per base N content

## Sequence Length Distribution

## Sequence Duplication Levels

## Overrepresented sequences

| Sequence | Count | Percentage | Possible Source |
| --- | --- | --- | --- |
| GTTTGATATTGGGATAGAGCTGGTGGTTTTATGTTAATGATTGTGGTAAT | 34559 | 0.13322023163489968 | No Hit |

## Kmer Content

| Sequence | Count | Obs/Exp Overall | Obs/Exp Max | Max Obs/Exp Position |
| --- | --- | --- | --- | --- |
| CTCCT | 13607090 | 3.6371665 | 5.25997 | 1 |
| CTTCT | 13250410 | 3.3235593 | 5.902106 | 6 |
| CTCCA | 10445710 | 3.3117285 | 5.9472055 | 6 |
| TCCTC | 12149310 | 3.2475028 | 5.2664833 | 2 |
| CAGCT | 10613580 | 3.150422 | 5.4491186 | 9 |
| TCCAG | 10415970 | 3.0917656 | 6.1420107 | 7 |
| TCTTC | 11284775 | 2.8305252 | 5.4218497 | 5 |
| CTTCA | 9046650 | 2.6914153 | 5.73313 | 6 |
| GCTCC | 8572330 | 2.286182 | 6.8759727 | 1 |
| TCCAC | 6651405 | 2.1087744 | 5.590794 | 2 |
| GCCAG | 7078985 | 2.096485 | 9.340548 | 1 |
| GCTGG | 8405180 | 1.9648935 | 10.510148 | 1 |
| GGCAG | 6987420 | 1.937437 | 5.357939 | 1 |
| GCTTC | 7672520 | 1.9201101 | 6.453423 | 1 |
| CTGGA | 6646175 | 1.8470075 | 5.2237854 | 2 |
| GGAGA | 5595920 | 1.72694 | 6.3880115 | 1 |
| TTTTT | 7815380 | 1.7261425 | 7.8898697 | 2 |
| GCCCA | 5226490 | 1.6532587 | 5.702715 | 1 |
| GGGAG | 6066340 | 1.574808 | 5.371084 | 1 |
| GGGGG | 6943895 | 1.5163487 | 7.1249323 | 1 |
| GGGGA | 5834290 | 1.5145683 | 7.931002 | 1 |
| GCCAC | 4779140 | 1.5117517 | 5.5515676 | 1 |
| GTCCA | 5069580 | 1.5048002 | 9.766185 | 1 |
| GCTCA | 4937460 | 1.4655832 | 6.493982 | 1 |
| GCTGA | 5089190 | 1.4143132 | 5.6804657 | 1 |
| GTCAG | 5019560 | 1.3949627 | 6.487554 | 1 |
| GGGAA | 4424485 | 1.365427 | 5.1437864 | 1 |
| GTCCT | 5424880 | 1.3576198 | 5.0257454 | 1 |
| GTTGA | 5199360 | 1.3558854 | 6.7676234 | 1 |
| GCCTG | 5390320 | 1.3459115 | 5.2206874 | 1 |
| GTGGG | 5980810 | 1.3090074 | 5.36666 | 1 |
| GTTGG | 5370765 | 1.1781602 | 7.2866774 | 1 |
| GTGGA | 4460710 | 1.1606225 | 5.391923 | 1 |
| GTTCA | 3889400 | 1.0833421 | 5.6475687 | 1 |
| GTTTT | 4793200 | 1.0562482 | 5.9062552 | 1 |
| GTTTG | 4119050 | 0.9056312 | 7.673619 | 1 |
| GTTTA | 2590030 | 0.6769616 | 5.639357 | 1 |

Produced by FastQC (version 0.10.1)
