# Supplementary material for: Meat Quality Characteristics of Mongolian Horses in Inner Mongolia: Regional Superiority and Transcriptomic Insights into Tenderness Differences Between Muscular Locations
Source: Animals (Basel). 2026 Jun 9;16(12):1788. doi: 10.3390/ani16121788 (PMC13295530; doi:10.3390/ani16121788)
Supplement: Supplementary file 1 [file animals-16-01788-s001.zip › TB7 R2.fq_fastqc.zip!/H15_TB_Clean_Data2.fq_fastqc/fastqc_report.html]

H15\_TB\_Clean\_Data2.fq.gz FastQC Report


FastQC Report

Thu 17 Dec 2020  
H15\_TB\_Clean\_Data2.fq.gz

## Summary

- Basic Statistics
- Per base sequence quality
- Per sequence quality scores
- Per base sequence content
- Per base GC content
- Per sequence GC content
- Per base N content
- Sequence Length Distribution
- Sequence Duplication Levels
- Overrepresented sequences
- Kmer Content

## Basic Statistics

| Measure | Value |
| --- | --- |
| Filename | H15\_TB\_Clean\_Data2.fq.gz |
| File type | Conventional base calls |
| Encoding | Sanger / Illumina 1.9 |
| Total Sequences | 25941255 |
| Filtered Sequences | 0 |
| Sequence length | 92-141 |
| %GC | 51 |

## Per base sequence quality

## Per sequence quality scores

## Per base sequence content

## Per base GC content

## Per sequence GC content

## Per base N content

## Sequence Length Distribution

## Sequence Duplication Levels

## Overrepresented sequences

No overrepresented sequences

## Kmer Content

| Sequence | Count | Obs/Exp Overall | Obs/Exp Max | Max Obs/Exp Position |
| --- | --- | --- | --- | --- |
| AGGAG | 13786835 | 3.506525 | 3.9760315 | 2 |
| AGAAG | 13294765 | 3.238861 | 5.2461715 | 3 |
| GAGGA | 12287840 | 3.1252728 | 6.0469785 | 1 |
| TGGAG | 10294810 | 3.1228244 | 5.037883 | 3 |
| AGCTG | 10612760 | 3.0946143 | 4.626099 | 6 |
| CTGGA | 10321340 | 3.0096374 | 6.360375 | 2 |
| GCTGG | 9478350 | 2.8854373 | 10.327872 | 1 |
| GGAGG | 10630250 | 2.8226469 | 5.4597836 | 1 |
| TGAAG | 8806465 | 2.5587633 | 5.2286687 | 3 |
| GGAGA | 9718905 | 2.471893 | 8.703731 | 1 |
| GCTGC | 8066940 | 2.3606772 | 5.650538 | 1 |
| GCTGA | 8038960 | 2.3441098 | 11.466266 | 1 |
| GAGAA | 8946860 | 2.1796274 | 5.3147593 | 2 |
| GTGGA | 6617440 | 2.007332 | 7.8478074 | 1 |
| CTGAA | 6997585 | 1.9544544 | 6.288329 | 2 |
| TTTGA | 4641825 | 1.837597 | 5.3793063 | 2 |
| GGAAG | 7196555 | 1.830362 | 5.669999 | 1 |
| GCAGA | 7472500 | 1.8269523 | 6.208136 | 1 |
| GCTGT | 4528795 | 1.5749896 | 5.0718017 | 1 |
| GCCAA | 6341810 | 1.4904705 | 6.756539 | 1 |
| GCTCT | 4427870 | 1.4802625 | 5.249641 | 1 |
| GTTTT | 2972865 | 1.4036331 | 5.3368025 | 1 |
| GCCAG | 5695810 | 1.3975469 | 6.371314 | 1 |
| GTTTG | 3197010 | 1.3213147 | 8.628783 | 1 |
| GCTCA | 4702850 | 1.3182207 | 5.1056304 | 1 |
| GTTCA | 3162340 | 1.053423 | 7.8200355 | 1 |
| GTTGA | 2359675 | 0.81770664 | 5.9756894 | 1 |
| GTTTA | 1911010 | 0.75652707 | 5.133276 | 1 |

Produced by FastQC (version 0.10.1)
