# Supplementary material for: Meat Quality Characteristics of Mongolian Horses in Inner Mongolia: Regional Superiority and Transcriptomic Insights into Tenderness Differences Between Muscular Locations
Source: Animals (Basel). 2026 Jun 9;16(12):1788. doi: 10.3390/ani16121788 (PMC13295530; doi:10.3390/ani16121788)
Supplement: Supplementary file 1 [file animals-16-01788-s001.zip › TB8 R1.fq_fastqc.zip!/H18_TB_Clean_Data1.fq_fastqc/fastqc_report.html]

H18\_TB\_Clean\_Data1.fq.gz FastQC Report


FastQC Report

Thu 17 Dec 2020  
H18\_TB\_Clean\_Data1.fq.gz

## Summary

- Basic Statistics
- Per base sequence quality
- Per sequence quality scores
- Per base sequence content
- Per base GC content
- Per sequence GC content
- Per base N content
- Sequence Length Distribution
- Sequence Duplication Levels
- Overrepresented sequences
- Kmer Content

## Basic Statistics

| Measure | Value |
| --- | --- |
| Filename | H18\_TB\_Clean\_Data1.fq.gz |
| File type | Conventional base calls |
| Encoding | Sanger / Illumina 1.9 |
| Total Sequences | 27158747 |
| Filtered Sequences | 0 |
| Sequence length | 92-141 |
| %GC | 51 |

## Per base sequence quality

## Per sequence quality scores

## Per base sequence content

## Per base GC content

## Per sequence GC content

## Per base N content

## Sequence Length Distribution

## Sequence Duplication Levels

## Overrepresented sequences

| Sequence | Count | Percentage | Possible Source |
| --- | --- | --- | --- |
| GTTTCATGTTGTATATGCGTCTGGATAATCAGAATAGCGTCGTGGCATTC | 32400 | 0.11929858177919621 | No Hit |

## Kmer Content

| Sequence | Count | Obs/Exp Overall | Obs/Exp Max | Max Obs/Exp Position |
| --- | --- | --- | --- | --- |
| CTCCT | 14226665 | 3.5876615 | 5.184793 | 2 |
| CTTCT | 13902830 | 3.30433 | 5.9014344 | 6 |
| TCCTC | 13068995 | 3.2957215 | 7.005334 | 2 |
| CTCCA | 10714950 | 3.2369304 | 6.2404733 | 1 |
| CAGCT | 10487175 | 2.967763 | 5.2371306 | 9 |
| TCCAG | 10363830 | 2.932858 | 6.9413314 | 2 |
| TCTTC | 12120235 | 2.880655 | 5.847935 | 5 |
| CTTCA | 9600850 | 2.7335362 | 6.235449 | 6 |
| TTCTT | 11418835 | 2.557843 | 5.1283236 | 6 |
| GCTCC | 8802215 | 2.206256 | 7.583931 | 1 |
| TCCAC | 7187055 | 2.1711717 | 6.5955834 | 2 |
| GCCAG | 7622595 | 2.144022 | 8.709029 | 1 |
| GGCAG | 7459910 | 1.9655653 | 6.63742 | 1 |
| GCTGG | 8730830 | 1.9203242 | 9.273944 | 1 |
| GCTTC | 8044910 | 1.9004525 | 6.03152 | 1 |
| GGAGA | 5677075 | 1.6888279 | 5.3157067 | 1 |
| GCCCA | 5560600 | 1.669631 | 5.3180723 | 1 |
| GGGAG | 6347135 | 1.5666039 | 5.3906283 | 1 |
| GTCCA | 5448415 | 1.5418459 | 12.524889 | 1 |
| GGGGG | 7424365 | 1.5204128 | 6.403253 | 1 |
| GGGGA | 6131380 | 1.5133513 | 7.161645 | 1 |
| GCCAC | 5028830 | 1.509961 | 5.151844 | 1 |
| TTTTT | 7015510 | 1.4810964 | 6.1121373 | 2 |
| GGGAA | 4770250 | 1.4190637 | 5.772775 | 1 |
| GTTGA | 5604375 | 1.4002237 | 8.802289 | 1 |
| GTCAG | 5261030 | 1.3946625 | 9.814541 | 1 |
| GCTCA | 4877385 | 1.3802501 | 5.4607487 | 1 |
| GTCCT | 5819770 | 1.3748069 | 8.355059 | 1 |
| GTGGG | 6542210 | 1.3479415 | 7.715178 | 1 |
| GTCAT | 4513715 | 1.2038624 | 6.0552893 | 1 |
| GTTCT | 5368370 | 1.1952266 | 5.1975756 | 1 |
| GTCTG | 5294675 | 1.1716632 | 5.8521647 | 1 |
| GTGGA | 4672245 | 1.1602498 | 7.221667 | 1 |
| GTTCA | 4301375 | 1.1472287 | 5.55265 | 1 |
| GTTGG | 5501585 | 1.1404567 | 9.622723 | 1 |
| GTGCT | 5140770 | 1.1376053 | 5.9561257 | 1 |
| GTCAA | 3447070 | 1.1013559 | 5.306661 | 1 |
| GTTTT | 4881650 | 1.0243453 | 6.1205654 | 1 |
| GTCAC | 3569370 | 1.0100954 | 6.127394 | 1 |
| GTTTG | 4596765 | 0.9587109 | 7.2077236 | 1 |
| GTGCA | 3408265 | 0.90350735 | 5.3352714 | 1 |
| GTTTA | 2608660 | 0.6557406 | 5.2538185 | 1 |

Produced by FastQC (version 0.10.1)
