# Supplementary material for: Meat Quality Characteristics of Mongolian Horses in Inner Mongolia: Regional Superiority and Transcriptomic Insights into Tenderness Differences Between Muscular Locations
Source: Animals (Basel). 2026 Jun 9;16(12):1788. doi: 10.3390/ani16121788 (PMC13295530; doi:10.3390/ani16121788)
Supplement: Supplementary file 1 [file animals-16-01788-s001.zip › TB8 R2.fq_fastqc.zip!/H18_TB_Clean_Data2.fq_fastqc/fastqc_report.html]

H18\_TB\_Clean\_Data2.fq.gz FastQC Report


FastQC Report

Thu 17 Dec 2020  
H18\_TB\_Clean\_Data2.fq.gz

## Summary

- Basic Statistics
- Per base sequence quality
- Per sequence quality scores
- Per base sequence content
- Per base GC content
- Per sequence GC content
- Per base N content
- Sequence Length Distribution
- Sequence Duplication Levels
- Overrepresented sequences
- Kmer Content

## Basic Statistics

| Measure | Value |
| --- | --- |
| Filename | H18\_TB\_Clean\_Data2.fq.gz |
| File type | Conventional base calls |
| Encoding | Sanger / Illumina 1.9 |
| Total Sequences | 27158747 |
| Filtered Sequences | 0 |
| Sequence length | 92-141 |
| %GC | 51 |

## Per base sequence quality

## Per sequence quality scores

## Per base sequence content

## Per base GC content

## Per sequence GC content

## Per base N content

## Sequence Length Distribution

## Sequence Duplication Levels

## Overrepresented sequences

No overrepresented sequences

## Kmer Content

| Sequence | Count | Obs/Exp Overall | Obs/Exp Max | Max Obs/Exp Position |
| --- | --- | --- | --- | --- |
| AGGAG | 14489735 | 3.4593449 | 4.5229797 | 2 |
| AGAAG | 14032105 | 3.2066271 | 5.3334565 | 3 |
| GAGGA | 13271740 | 3.1685553 | 6.6238375 | 1 |
| TGGAG | 10580565 | 3.051033 | 4.3607297 | 7 |
| GAAGA | 12361365 | 2.8248284 | 6.2046285 | 2 |
| GGAGG | 11025120 | 2.7499485 | 5.410707 | 1 |
| GCTGG | 9383470 | 2.7279053 | 6.532539 | 1 |
| TTTTT | 4761150 | 2.562388 | 5.5170026 | 2 |
| GGAGA | 10330580 | 2.4663694 | 8.8226385 | 1 |
| GCTGA | 8188870 | 2.2786727 | 7.4671273 | 1 |
| GAGAA | 9447465 | 2.1589415 | 5.7199225 | 2 |
| GTGGA | 7179140 | 2.0701914 | 8.598849 | 1 |
| CCAGC | 8679585 | 1.9453602 | 5.068627 | 8 |
| GGAAG | 7884675 | 1.8824229 | 6.616898 | 1 |
| TTTGA | 4810670 | 1.8541173 | 6.125722 | 2 |
| GCCAA | 6540025 | 1.453961 | 5.3481693 | 1 |
| GCCAG | 6053315 | 1.4059647 | 5.241729 | 1 |
| GTTTT | 2952765 | 1.3745646 | 7.5866404 | 1 |
| GGAAA | 6003125 | 1.3718385 | 6.10848 | 1 |
| GTTTG | 3323075 | 1.3380724 | 9.045342 | 1 |
| GTGAA | 4735815 | 1.3071485 | 6.169559 | 1 |
| GTTCT | 2836210 | 1.1020411 | 6.0254264 | 1 |
| GTTCA | 3237575 | 1.0415368 | 6.7237306 | 1 |
| GTTGA | 2534085 | 0.8448041 | 6.3135333 | 1 |

Produced by FastQC (version 0.10.1)
